# Fecal microbiota transplantation for irritable bowel syndrome: a systematic review and meta-analysis of randomized controlled trials

**DOI:** 10.3389/fimmu.2023.1136343

**Published:** 2023-05-18

**Authors:** Mancai Wang, Xiaofeng Xie, Songbo Zhao, Xiaojuan Ma, Zheyuan Wang, Youcheng Zhang

**Affiliations:** ^1^ Department of General Surgery, Lanzhou University Second Hospital, Lanzhou, China; ^2^ Department of Histology and Embryology, Medical College of Northwest Minzu University, Lanzhou, China

**Keywords:** fecal microbiota transplantation, irritable bowel syndrome, systematic review, meta-analysis, gut microbiome, influence factor

## Abstract

**Objective:**

Whether fecal microbiota transplantation (FMT) in patients with irritable bowel syndrome (IBS) is effective in improving outcomes remains controversial. We assessed the safety and efficacy of FMT for patients with IBS.

**Methods:**

In this systematic review and meta-analysis, we searched PubMed, Embase, Web of Science, the Cochrane Library, the clinicaltrials.gov and International Clinical Trials Registry Platform (ICTRP) up to February 25, 2022, updated to March 28, 2023. Randomized controlled trials (RCTs) compared the stool and capsule FMT with placebo in patients with IBS were included. Two authors independently assessed study eligibility, extracted the data, and assessed risk of bias. We did meta-analysis with RevMan, and the Stata software was used for sensitivity analysis and meta-regression. The GRADE system was used to assess the quality of evidences. Mean difference (MD) or standardized Mean difference (SMD) with 95% CI for continuous data, and risk ratios (RR) with 95% CI for dichotomous data were used with random-effects models. The primary outcomes included the clinical response rate and IBS-SSS score. This study is registered with PROSPERO: CRD42022328377.

**Results:**

Nineteen reports from nine RCTs were included finally. Compared with the placebo, a single stool FMT could significantly decrease the IBS-SSS score at 1 month (MD=-65.75, 95%CI [-129.37, -2.13]), 3 months (MD=-102.11, 95% CI [-141.98, -62.24]), 6 months (MD=-84.38, 95%CI [-158.79, -9.97]), 24 months (MD=-110.41, 95%CI [-145.37, -75.46]), and 36 months (MD=-104.71, 95%CI [-137.78, -71.64]). It also could improve the clinical response rate at 3 months (RR=1.91, 95% [1.12, 3.25]), 24 months (RR=2.97, 95% [1.94, 4.54]), and 36 months (RR=2.48, 95% [1.65, 3.72]), and increase the IBS-QoL score at 3 months, 24 months, and 36 months. FMT did not increase the serious adverse event. The risk of bias was low, and the quality of evidence based on GRADE system was moderate in the stool FMT group. However, we did not find positive effect of capsule FMT on patients with IBS based on the current available data.

**Conclusion:**

A single stool FMT is effective and safe for patients with IBS. However, some factors may affect the effectiveness of FMT, and the relationship between the gut microbiome and the effect of FMT for IBS is still unclear.

**Systematic review registration:**

https://www.crd.york.ac.uk/prospero/, identifier CRD42022328377.

## Introduction

Irritable bowel syndrome (IBS) is one of the most common functional gastrointestinal disorders (FGIDs) which now called disorders of gut-brain interaction (1). The prevalence of IBS appears to vary widely between different countries all over the world, according to the latest research, the average varies between 9.2%-10.1% and 3.8%-4.1% used the Rome III criteria and the Rome IV criteria, respectively (1, 2). IBS is characterized by symptoms including recurrent abdominal pain associated with a change in stool form or frequency, it has resulted in significant global health care costs and impaired health-related quality of life (3–6).

IBS is difficult to treat and conventional therapies are often ineffective at controlling symptoms and restoring function (7). The pathophysiology of IBS is complex and incompletely understood up to now, it may associate with the altered gut-brain axis, stress, disordered gastrointestinal motility, abnormal intestinal secretion, visceral hypersensitivity, immunomodulation, and intestinal permeability, and all of these can be affected by the gut microbial community (3, 8). More and more researches show that gut microbiota dysbiosis plays an important role in IBS pathogenesis (9–11).

Fecal microbiota transplantation (FMT) is a non-conventional therapy in which fecal material from healthy donors is given to patients attempt to cure disease or relieve symptoms (7). It is an efficient way of modulating the gut microbiota and aims to introduce a balanced conglomerate of microorganisms (12). It has shown definite efficacy for the treatment of recurrent *Clostridioides difficile* infection (13, 14). In addition, it has also been used for some gastrointestinal diseases such as inflammatory bowel disease (15). FMT is being explored as a therapeutic option for the patients of IBS, positive effects on IBS symptoms in various degrees were obtained in some randomized controlled trials (RCTs), while there was no effect in the others, so the results from these RCTs are inconsistent (16).

So far, some meta-analyses have evaluated the efficacy of FMT in the treatment of IBS, and they unanimously concluded that FMT is ineffective for IBS (17–19). Unfortunately, some recently published RCTs (20–22) were not included in these analyses, so the conclusions may not represent the real results very well. We therefore conduct an updated meta-analysis and systematic review of RCTs to re-estimate the efficacy and safety of FMT for the treatment of IBS.

## Methods

The systematic review and meta-analysis was performed in accordance with the Cochrane Handbook for Systematic Reviews of Intervention (23) and the PRISMA statement (24). The study was registered in PROSPERO (CRD42022328377).

### Eligibility criteria

The PICOS (patients, intervention, comparison, outcomes, and study design) tool was used to specify eligibility criteria for the systematic review and meta-analysis (25, 26). Patients with moderate to severe IBS diagnosed according to the Rome III or IV criteria, aged ≥ 18 years, the subtypes of IBS were not restricted; allogenic FMT was used as the intervention, the routes, frequency and does were not restricted; autologous FMT or placebo capsules were used as comparison measures for patients in the placebo group; the main outcomes included clinical response rate, IBS-SSS score, IBS-QoL score, abdominal pain, frequency of stool, side effects and the change of microbiome profiles; only randomized controlled trials were included.

### Search strategy

We systematically searched the electronic databases PubMed, Embase, Web of Science, the Cochrane Library, the clinicaltrials.gov and International Clinical Trials Registry Platform up to February 25, 2022, updated to March 28, 2023. This search was performed using both free text and Mesh terms. Search terms included fecal, faecal, feces, faeces, stool, microbiota, microbiome, microflora, bacteria, transplantation, transplant, transfer, irritable bowel syndrome, IBS. The full search syntaxes were supplied in **Supplementary Table 1**.

### Study selection

The study screening and selection was performed in accordance with the PRISMA 2020 flow diagram (24) by a three-step process. In the first step, all database citations got by preliminary searching were imported and de-duplicated in EndNote (27). In the second step, titles, abstracts, and keywords of citations were screened separately by two authors (MCW and XFX) to identify potentially eligible studies. In the third step, the full texts of these potentially eligible studies were examined to identify the studies that ultimately met the eligibility criteria above. If consensus could not be reached, a third co-author (YCZ) provided input. In the case of multiple papers from the same RCTs, relevant data were extracted from all papers, they were included as a single study in the analysis and identified uniformly by the only register number.

### Data extraction

Two reviewers (MCW, XFX) independently extracted data from all full-text articles that met eligibility criteria in a prespecified Microsoft Excel spreadsheet, and any disagreement was resolved by discussion with a third co-author (YCZ). The means and standard deviations were collected for continuous variables, if they were not reported in the text, the data would be extracted from their plots, images, and maps using a web-based tool WebPlotDigitizer 4.5 (28, 29). When these data were not available and whenever possible, the 95% CIs and P values were used to calculate means and standard deviations using the RevMan Calculator which was provided in the Review Manager 5 (Version 5.4). Where sample size, median, range and/or interquartile range were reported, they were converted to means and standard deviations according to the conversion formulas of Wan et al. (30) and Shi et al. (31), which will often give an advantage over the omission of trials with missing means or standard deviations from a meta-analysis (32). Where insufficient data were available to calculate or extract the means and standard deviations, the study was excluded from quantitative analysis.

### Quality assessment

Two authors (MCW, XFX) independently assessed the quality of the systematic review and meta-analysis, disagreements were resolved by discussing with a third co-author (YCZ). The risk of bias of each included studies was assessed with the Cochrane Collaboration’s tool in the Cochrane Handbook for Systematic Reviews of Interventions (RevMan software, Version 5.4) (23). The quality of evidence for each outcome was assessed with the GRADE system (GRADEpro software, Version 3.6), the quality could be downgraded by one level (serious concern) or two levels (very serious concerns) due to these factors: risk of bias (33), inconsistency (34), indirectness (35), imprecision (36), and publication bias (37), the grade was specified four categories as high, moderate, low, and very low (38, 39).

### Outcomes and summary measures

The primary outcome was the IBS-SSS score at different time points after FMT. The secondary outcomes included the clinical response rate, IBS-QoL score, abdominal pain, frequency of stool, stool consistency, adverse events, and the change of microbiome profiles. The clinical response rate was defined by the relief level of IBS symptoms, and the symptoms were assessed using the IBS-severity scoring system (IBS-SSS) (40), or Gastrointestinal Symptom Rating Scale for IBS (GSRS-IBS) (41), or a daily symptom diary (22).

### Data synthesis and analysis

Data synthesis and analysis was performed using the RevMan software (Version 5.4). we reported data in terms of mean difference (MD) and 95% confidence interval (CI) for continuous data. When different studies used different rating instruments to measure the same outcome, the standardized mean difference (SMD) would be reported (42). For dichotomous data, we reported risk ratios (RR) and 95% CI. We identified heterogeneity from forest plots using the Chi (2) test with a significance level of p= 0.1. The heterogeneity was quantified using the I^2^ statistic, where I^2^ ≥ 50% indicated a significant heterogeneity (43). When the I^2^ ≥ 50%, we would assess the possible sources of heterogeneity using sensitivity analysis. All meta-analysis were performed used random-effects models considering the heterogeneous in terms of interventions, participant characteristics, donor characteristics, and outcome measurements among included studies. Where meta-analysis was not possible or appropriate, we would present results as qualitative synthesis of intervention effects.

Where sufficient data were available, we planned to perform subgroup analyses based on the stool FMT and capsule FMT, meta-regression analysis would also be performed for different routes, dose, frequency of FMT, number of donors, and for different style of stools. When the number of included studies was more than 10 (44), we would assess the publication bias for the outcomes of clinical response rate, IBS-SSS and IBS-QoL using the funnel plot and Egger’s test. The Stata software (Version 12) was used to assess the publication bias, sensitivity analysis and meta-regression analysis.

## Results

### Study selection and the characteristics

**Figure 1:**
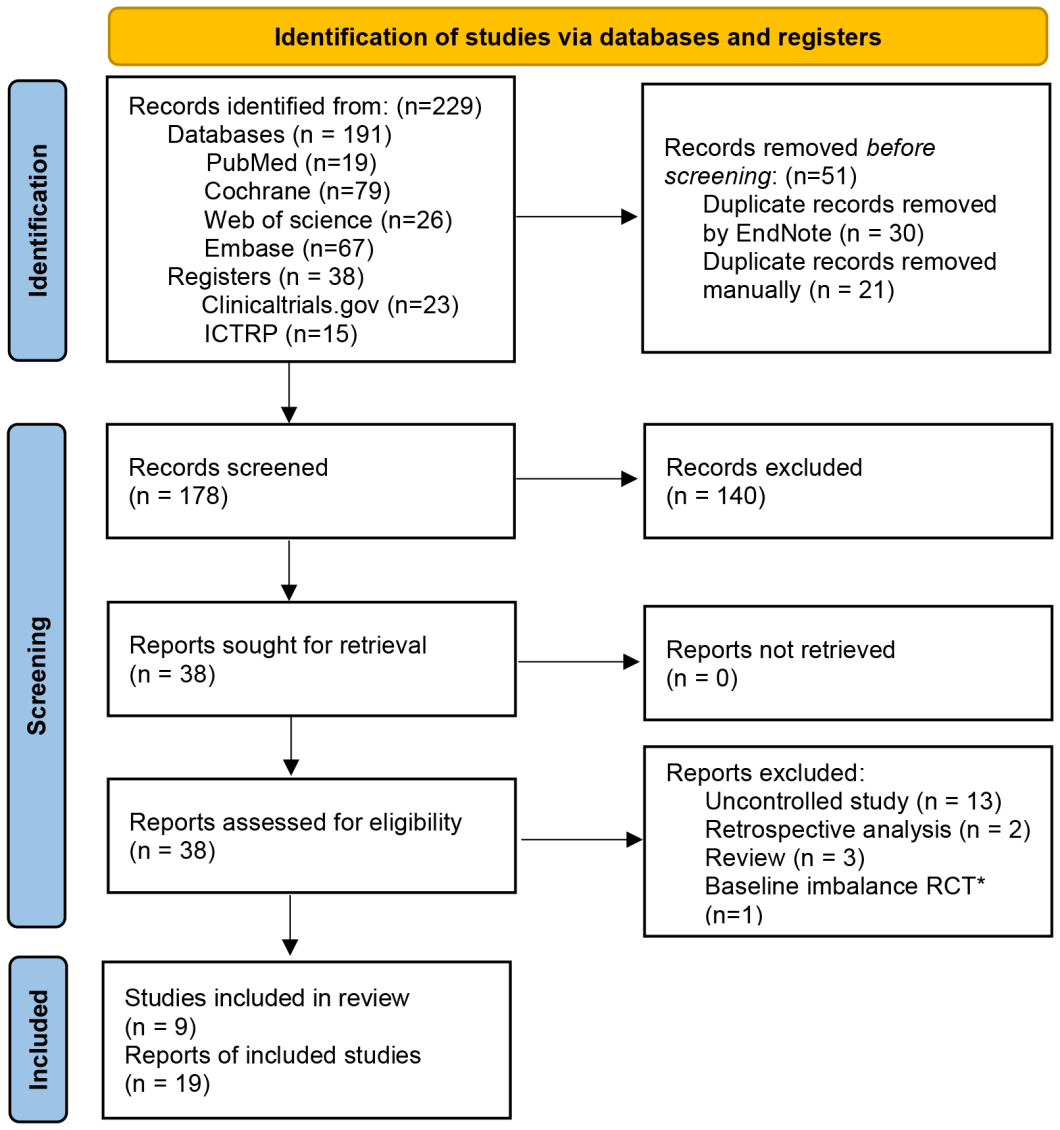
PRISMA 2020 flow diagram ICTRP, International Clinical Trials Registry Platform. *The study did not report the baseline data of patients for different groups in detail, IBS-SSS score in the transplantation day between FMT group and placebo group was significantly statistical difference (352.1±27 vs. 309.8±20).

Nineteen articles (7, 12, 20–22, 45–58) from nine eligible RCTs were included finally with a total sample size of 516. All the RCTs were registered in the clinicaltrials.gov or ICTRP. The flow chart of study selection was shown in the PRISMA 2020 flow diagram (24) (**Figure 1**). The characteristics of included studies were represented in **Table 1**. Of the nine included studies, two (7, 54) were conducted in the USA, one (55) in China, and the rest were in European countries. Patients with moderate to severe IBS symptoms were enrolled in the included studies. For the IBS subtypes, IBS-D (diarrhea-predominant IBS) and IBS-M (mixed-diarrhea-and constipation IBS) accounted for about 81.7% of patients in these studies in total. Diagnosis of IBS based on Rome III criteria in 8 RCTs and Rome IV criteria in one RCT (NCT03822299). The follow-up time varied between 10 weeks and 52 weeks. The main outcomes and design of these included studies were represented in **Supplementary Table 2**.

Table 1The baseline characteristics of included studies.

**Table:** 

Trial ID	Country	Year	Journal	Author	Sample size (FMT/Control)	Age	Sex (M: F)		Diagnostic criteria
							FMT	placebo	
NCT02299973	Belgium	2021	Gastroenterology	Holvoet T	62(43/19)	18-75	13/30	11/8	Rome III
NCT02788071	Denmark	2018	Gut	Halkjær SI	51(25/26)	18-60	8/17	8/18	Rome III
		2021	Scand J Gastroenterol	Madsen AMA					
		2021	Gut Microbes	Browne PD					
NCT03822299	Norway	2020	Gut	El-Salhy M	165(55/55/55)	18-85	14/40 46/9	8/47	Rome IV
		2021	World J Gastroenterol	El-Salhy M					
		2021	Neurogastroenterol Motil	El-Salhy M					
NCT03561519	Finland	2020	Aliment Pharmacol Ther	Lahtinen P	49(23/26)	18-73	12/11	17/9	Rome III
NCT02154867	Norway	2018	Lancet Gastroenterol Hepatol	Johnsen PH	83(55/28)	18-75	19/36	9/19	Rome III
		2020	EBioMedicine	Johnsen PH					
		2020	Gut Microbes	Goll R					
NCT02328547	USA	2019	Lancet Gastroenterol Hepatol	Aroniadis OC	48(25/23)	18-65	16/9	14/9	Rome III
NCT02092402	Sweden	2019	Clin Transl Gastroenterol	Holster S	17(8/9)	18-65	5/3	3/5	Rome III
		2019	Biomolecules	Holster S					
NCT02847481	USA	2022	Gut Microbes	Singh P	23(11/12)	18-80	5/6	7/5	Rome III
ChiCTR1900024924	China	2021	Microb Cell Fact	Lin H	18(9/9)	18-80	5/4	5/4	Rome III

**Table:** 

Trial ID	IBS-SSS score		IBS-QoL score		IBS subtypes (FMT/placebo)	Follow-up time		Lost to follow-up	
	FMT	placebo	FMT	placebo				FMT	placebo
NCT02299973	380(270-390)	370(310-440)	32.6(11-119)	29.1 (22-61)	IBS-D IBS-M	12 weeks 1 year		0/43	1/19
NCT02788071	341.68±95.02	345.04±79.56	42.07±14.75	40.11±15.42	IBS-C:7/10 IBS-D:7/8 IBS-M:11/8	1, 3, 6 months		1/26	0/26
NCT03822299	311.8±76.8 313.9±87.3	315.2±77.1	109.1±22.7 113.4±22.4	117.8±19.7	IBS-C:20/20/22 IBS-D:22/20/21 IBS-M:13/14/12	2 weeks 1, 3 months		1/110	0/55
NCT03561519	282.5±85.4	263.5 (93.2)	56.9±19.9	57.2±20.3	IBS-D:9/16 IBS-M:3/4 IBS-other:11/6	4, 8, 12, 26, 52 weeks		4/27	2/28
NCT02154867	260(226-313)	278(223-254)	57.7±19.1	49.2±20.6	IBS-D:31/13 IBS-M:24/15	3, 12 months		2/57	2/30
NCT02328547	282±65	309±64	53±18	52±18	IBS-D:25/23	12 weeks 24 weeks		3/25	0/23
NCT02092402	No reported	No reported	No reported	No reported	IBS-C:1/3 IBS-D:5/4 IBS-M:2/1	2, 4, 8 weeks 6 months		0/8	2/9
NCT02847481	347.5±59.0	282.3±70.7	42.7±19.2	47.6±13.4	IBS-D: 11/12	1, 10 weeks		3/11	1/12
ChiCTR1900024924	291.11±42.28	284.44±40.86	43.33±7.53	44.11±7.61	IBS-D: 9/9	1 week 1, 2, 3 months		0/9	0/9

### FMT characteristics

The characteristics of FMT in these included RCTs were represented in **Table 2**. The styles of FMT materials included fresh or frozen donor stool and fecal microbiota capsule in the FMT group. In the placebo group, the placebo materials included autologous stool and placebo capsule. The route of FMT administration included nasojejunal probe, gastroscope, colonoscopy and oral capsules. In the stool FMT group, the dose of fresh stool was 30g, 50g, 60g, and 50-80g, and in the capsule FMT group, the dose of fresh stool was 14.25g, 28.5g and 600g. Two RCTs (45, 55) did not reported the dose of stool. All patients were given just a single FMT in the stool FMT group, a second FMT was offered only in one study (45) after the cross-over part in the capsule FMT group.

**Table 2:** The characteristics of FMT in these included RCTs.

Trial ID	Intervening measure		Route of FMT (position)	Frequency of FMT	Number of donor	Style of stools	Dose of stool	Effective for IBS
	FMT	placebo						
NCT02299973	non-capsule FMT	autologous stools	nasojejunal tube (jejunum)	single, second	2	fresh, from 1 donor	no report	Yes
NCT02788071	capsule FMT	placebo capsules	oral capsules (stomach)	12 days	4	frozen, mixed	600g (50g/day×12 days)	No
NCT03822299	non-capsule FMT	autologous stools	gastroscope (distal duodenum)	single	1	frozen, from 1 donor	30g/60g	Yes
NCT03561519	non-capsule FMT	autologous stools	colonoscopy (caecum)	single	1	frozen, from 1 donor	30g	Yes
NCT02154867	non-capsule FMT	autologous stools	colonoscopy (caecum)	single	2	fresh/frozen, mixed	50-80g	Yes
NCT02328547	capsule FMT	placebo capsules	oral capsules (stomach)	3 days	4	frozen, from 1 donor	28.5g (9.5g/day×3 days)	No
NCT02092402	non-capsule FMT	autologous stools	colonoscopy (caecum)	single	2	frozen, from 1 donor	30g	Yes
NCT02847481	capsule FMT	placebo capsules	oral capsules (stomach)	1 day	6	frozen, mixed	14.25g (14.25g/d×1day)	No
ChiCTR1900024924	capsule FMT	placebo capsules	oral capsules (stomach)	3 days	1	frozen, from 1 donor	no report (30 capsules/day×3 days)	Yes

NCT, national clinical trial; FMT, fecal microbiota transplantation; M, male; F, female; IBS-SSS, irritable bowel syndrome severity scoring system; IBS-QoL, irritable bowel syndrome specific quality of life.

### Risk of bias

**Figure 2:**
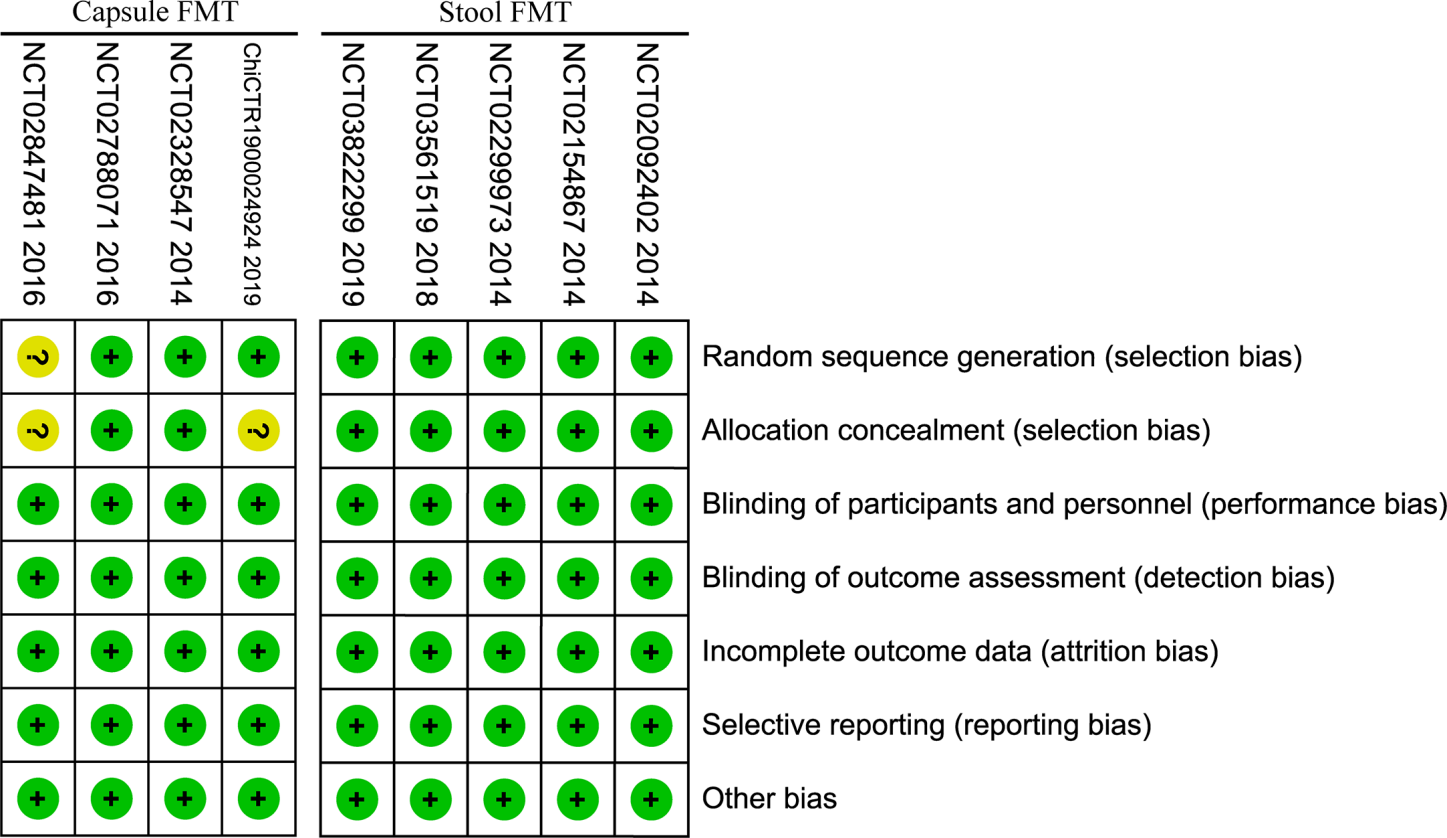
Risk of bias of included studies.

Risk of bias assessment showed low to moderate risk for all the included studies (**Figure 2**). Randomized controlled design was performed in all included studies, and the allocation concealment for the random sequences was used in seven RCTs. Meanwhile, study participants and investigators were blinded to treatment allocation. Loss of follow-up was reported in detail in all studies and managed appropriately. The number of lost to follow-up was similar between FMT group and placebo group, there was no significant difference in the meta-analysis (RR=1.30, 95% [0.56, 3.01]).

### Efficacy of FMT for IBS

An important feature of partially included studies (20, 22) was that some of the outcomes in the FMT group changed significantly from baseline at the end of the intervention, which did not occur in the placebo group, but there were no statistically significant differences between the FMT group and placebo group at the end of the intervention. Therefore, in order to comprehensively analyze the effect of FMT on IBS patients, we not only vertically analyzed the differences of these outcomes between the two groups after the end of intervention, but also horizontally analyzed the differences between the baseline and endpoint after intervention in the two groups, separately.

### FMT group versus placebo group at different time points

#### IBS-SSS score

Four RCTs (20, 21, 46, 55) reported the IBS-SSS score at 1 month/4weeks, one (54) reported at 10 weeks, six (7, 20, 21, 46, 50, 55) at 3 months/12 weeks, two (46, 50) at 6 months, one (20) at 52 weeks, one (57) at 24 months, and one (57) at 36 months. Meta-analysis with random-effects models shown that there were statistically significant differences between FMT and placebo groups at 1 month, 3 months, 24months and 36 months (1 month: MD=-55.72, 95%CI [-105.01, -6.43]; 3 months: MD=-69.60, 95%CI [-98.09, -41.12]; 24 months: MD=-110.41, 95%CI [-145.37, -75.46]; 36 months: MD=-104.71, 95%CI [-137.78, -71.64]), but there were not statistically significant differences at other time points (10 weeks: MD=61.10, 95%CI [-30.86, 153.06]; 6 months: MD =-27.87, 95%CI [-138.28, 82.54]; 52 weeks: MD =-12.68, 95%CI [-82.76, 57.40]) (**Supplementary Figure 2**).

**Figure 3:**
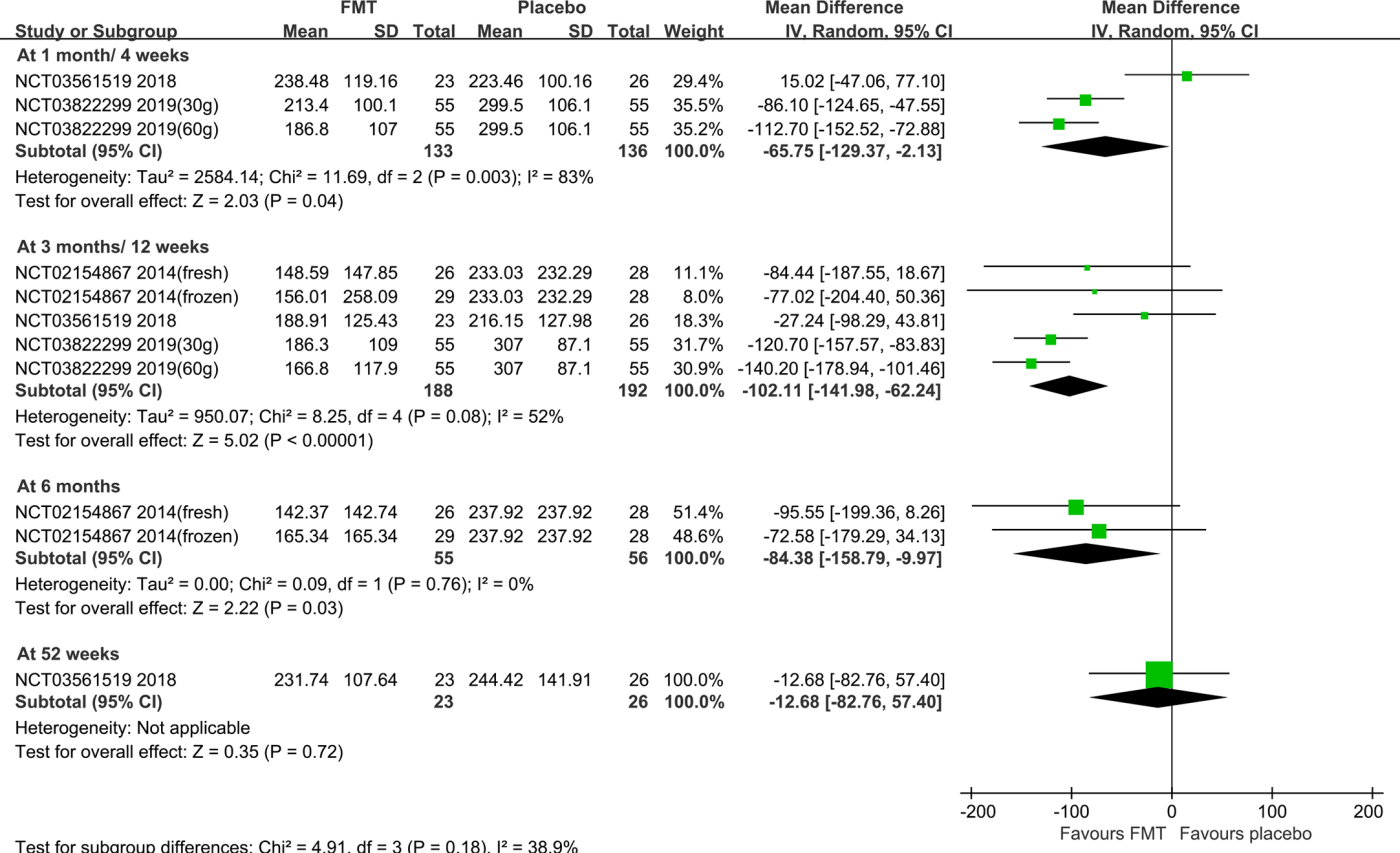
IBS-SSS score in the stool FMT group at different times.

Subgroup analysis based on the stool and capsule FMT shown that, compared with the placebo group, there were statistically significant differences in the stool FMT group at 1 month (MD=-65.75, 95%CI [-129.37, -2.13]), 3 months (MD=-102.11, 95%CI [-141.98, -62.24]), 6 months (MD=-84.38, 95%CI [-158.79, -9.97]), 24 months (MD=-110.41, 95%CI [-145.37, -75.46]), and 36 months (MD=-104.71, 95%CI [-137.78, -71.64]) (**Figure 3**). Significant heterogeneity existed among these studies at both 1 month and 3 months. After sensitivity analysis, we respectively removed the obviously heterogeneous study Lahtinen et al. (NCT03561519) (20) at 1 month and at 3 months, the results were consistent with that before (**Supplementary Figure 3**). The differences were not statistically significant in the capsule FMT group compared with the placebo group at 1 month, 10 weeks, 3 months, or 6 months (p>0.05) (**Supplementary Figure 4**).

#### Clinical response rate

Five RCTs (7, 20, 21, 45, 50) reported the clinical response rate at 3 months/12 weeks, one (54) reported at 10 weeks, one (22) at 6 months, one (50) at 12 months, one (57) at 24 months, and one (57) at 36 months. Meta-analysis with random-effects models shown that there were not statistically significant differences between FMT and placebo groups at any time points (10 weeks: RR=0.39, 95% [0.11, 1.41]; 3 months: RR=1.60, 95%CI [0.92, 2.78]; 6 months: RR=4.00, 95% [0.56, 28.40]; 12 months: RR=1.58, 95%CI [0.91, 2.73]) except at 24 months (RR=2.97, 95% [1.94, 4.54]) and 36 months (RR=2.48, 95% [1.65, 3.72]) (**Supplementary Figure 1**).

**Figure 4:**
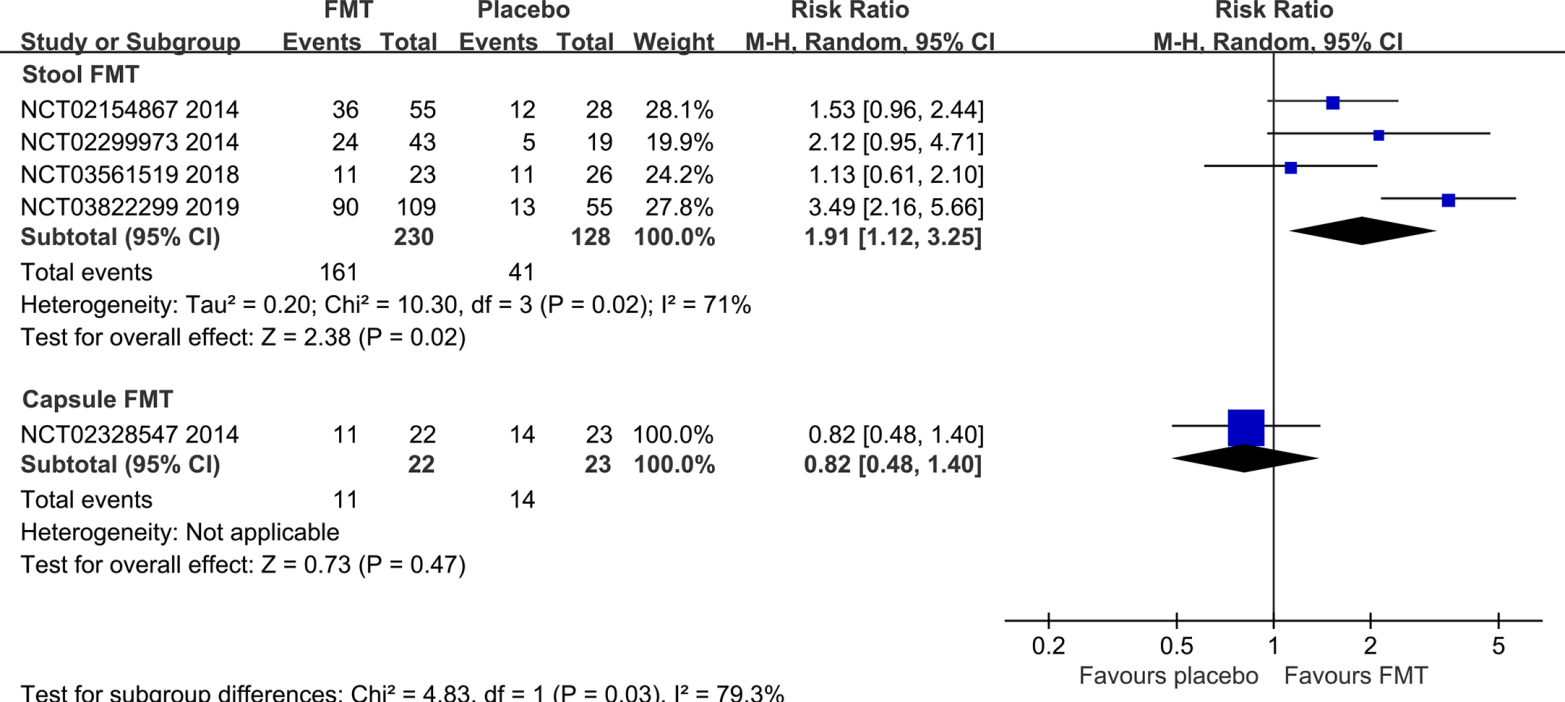
Subgroup analysis of clinical response rate in the stool and capsule FMT at 3 months.

Subgroup analysis based on the stool and capsule FMT shown that the clinical response rate in stool FMT group was significantly improved at 3 months/12 weeks compared with the placebo group (four RCTs, RR=1.91, 95% [1.12, 3.25]) (**Figure 4**). The difference was not statistically significant in the capsule FMT group compared with the placebo group (1 RCT, RR=0.82, 95%CI [0.48, 1.40]) (**Figure 4**). The average clinical response rate at 3 months with different definition was 70.0% (161/230, 4 RCTs) in the stool FMT group and 32.0% (41/128, 4 RCTs) in the placebo group. However, it should be emphasized that the definition of clinical response rate is not same in different studies (**Supplementary Table 3**). Significant heterogeneity existed among these studies (Chi^2 = ^10.30, I^2 = ^71%). After sensitivity analysis, we removed the obviously heterogeneous study El-Salhy et al. (NCT03822299) (21), the result was consistent with the previous one (Chi^2 = ^1.56, I^2 = ^0%; RR=1.48, 95%CI [1.06, 2.08]).

#### IBS-QoL score

Two RCTs (21, 46) with three pairs of data reported the IBS-QoL score at 1 month/4weeks, one RCT (54) reported at 10 weeks, four RCTs (7, 21, 45, 46) with five pairs of data reported at 3 months/12 weeks, one RCT (57) with two pairs of data reported at 24 months and 36 months. Meta-analysis shown that there were not statistically significant differences between FMT and placebo groups at 1 month/4 weeks (SMD=0.14, 95%CI [-0.11, 0.38]) and 10 weeks (SMD=0.30, 95%CI [-0.53, 1.12]), but there was statistically significant difference at 3 months/12 weeks, 24 months and 36 months (3months: SMD=0.62, 95%CI [0.33, 0.90]; 24 months: SMD=0.85, 95%CI [0.37, 1.33]; 36 months: SMD=1.07, 95%CI [0.67, 1.46]) (**Supplementary Figure 5**).

**Figure 5:**
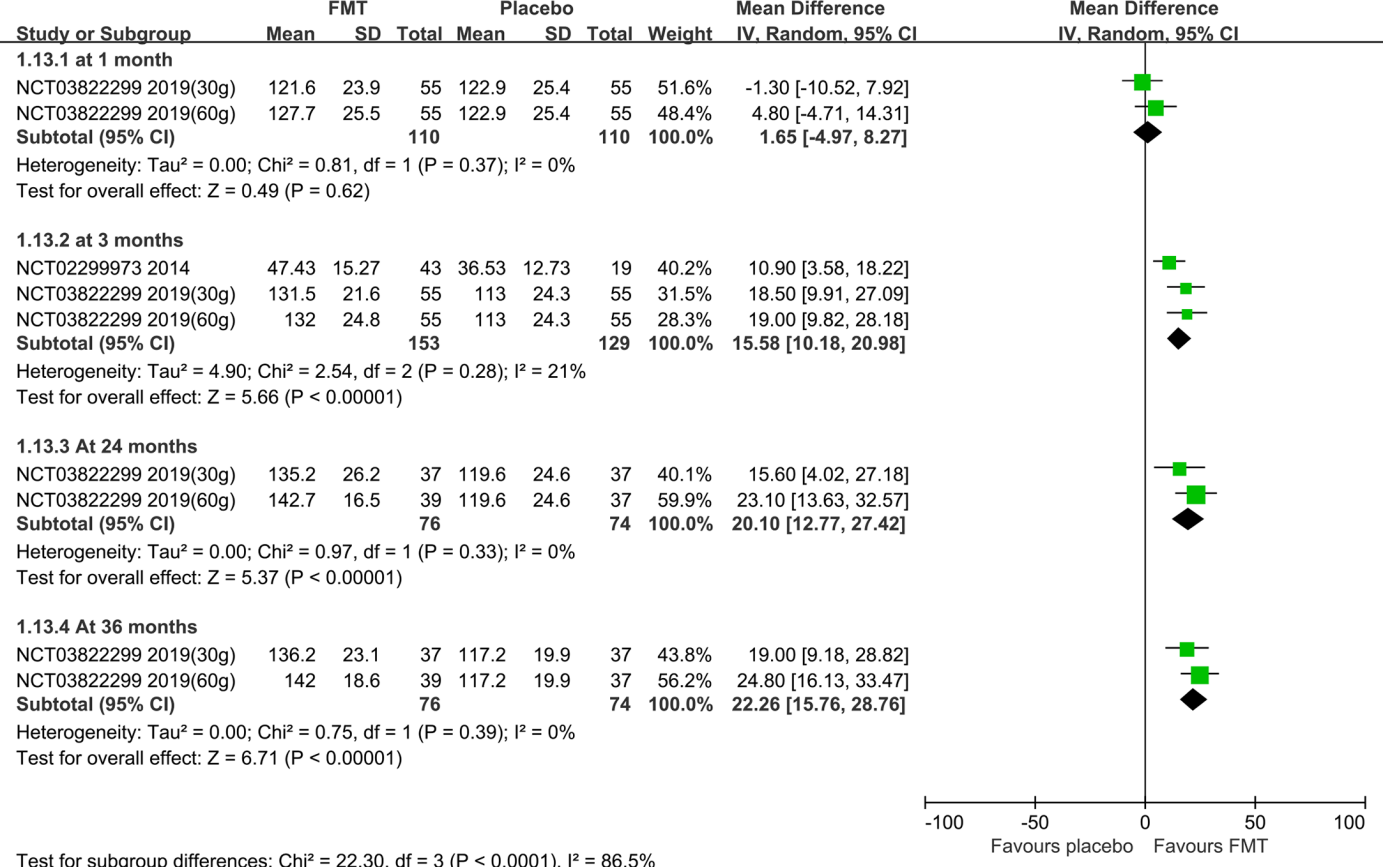
Subgroup analysis of IBS-QoL score in the stool and capsule FMT at 3 months.

Subgroup analysis shown that, compared with the placebo group, there were statistically significant differences in the stool FMT group at 3 months (SMD=0.78, 95%CI [0.53, 1.02]), 24 months (SMD=0.85, 95%CI [0.37, 1.33]), and 36 months (SMD=1.07, 95%CI [0.67, 1.46]) (**Figure 5**). However, there was no significant difference between capsule FMT group and placebo group at 1 months or 3 months.

#### Abdominal pain

The subgroup analysis was performed based on the stool FMT and capsule FMT for the abdominal pain at 3 months. Two RCTs (21, 45) with three pairs of data reported the abdominal pain in the stool FMT group, meta-analysis shown that there was significantly statistical difference compared with the placebo group (Chi^2 = ^0.05, I^2 = ^0%, SMD=-0.60, 95%CI [-0.84, -0.35]). One RCT (46) reported the outcome in the capsule group, there was no difference compared with the placebo group (SMD=0.38, 95%CI [-0.17, 0.93]).

#### Frequency of stool

The frequency of stool was reported in one RCT each in the stool FMT group (45) and capsule FMT group (46) at 3 months. Compared with the placebo group, there was significantly statistical difference in the stool FMT group (MD=-0.50, 95%CI [-0.93, -0.07]), while there was no difference in the capsule FMT group (MD=0.02, 95%CI [-0.63, 0.67]).

#### Stool consistency

The stool consistency was reported in one RCT each in the stool FMT group (45) and capsule FMT group (46) at 3 months. Compared with the placebo group, there was significantly statistical difference in the stool FMT group (MD=-0.33, 95%CI [-0.61, -0.05]), while there was no difference in the capsule FMT group (MD=0.06, 95%CI [-0.62, 0.44]).

### FMT group and placebo group versus their baseline

#### IBS-SSS score

In both the donor stool FMT group (FMT group) and the autologous stool FMT group (placebo group), two RCTs (20, 21) reported IBS-SSS score at baseline and at 1 month and 3 months after FMT, and one RCT (57) reported at 24 months and 36 months. Meta-analysis shown that, compared with its baseline, IBS-SSS score were significantly reduced at 1 month, 3 months, 24 months, and 36 months in the donor stool FMT group (1month: MD=-101.72, 95%CI [-124.49, -78.95]; 3 months: MD=-129.01, 95%CI [-153.05, -104.97]; 24 months: MD=-156.95, 95%CI [-188.41, -125.49]; 36 months: MD=-150.70, 95%CI [-179.91, -121.49]). But there were no statistical differences at 1 month (MD=-23.07, 95%CI [-52.01, 5.87]), 3 months (MD=-18.60, 95%CI [-52.50, 15.29]), 24 months (MD=-28.60, 95%CI [-70.01, 12.81]), or 36 months (MD=-27.70, 95%CI [-68.91, 13.51]) after FMT in the autologous stool FMT group.

In both the fecal microbiota capsule FMT group (FMT group) and the placebo capsule FMT group (placebo group), two RCT (46, 55) reported IBS-SSS score at baseline and at 1 month, three RCTs (7, 46, 55) reported IBS-SSS score at baseline and at 3 months, one RCT (46) reported it at 6 months after FMT. Meta-analysis shown that, compared with its baseline in the fecal microbiota capsule FMT group, IBS-SSS score were significantly reduced at 1 month and 3 months after FMT (MD=-102.66, 95%CI [-158.41, -46.91]; MD=-82.69, 95%CI [-126.74, -38.63]), while there was no difference between baseline and 6 months (MD=-43.95, 95%CI [-107.25, 19.35]). Surprisingly, in the placebo capsule FMT group, IBS-SSS score were also significantly reduced at 3 months and 6 months after FMT in the placebo capsule FMT group when compared with its baseline (MD=-66.92, 95%CI [-117.31, -16.53]; MD=-114.34, 95%CI [-171.73, -56.95]).

#### IBS-QoL score

In both the donor stool FMT group (FMT group) and the autologous stool FMT group (placebo group), one RCT (21) reported IBS-QoL score at baseline and at 1 month and 3 months after FMT, one RCT (45) reported it at baseline and at 3 months after FMT, and one RCT (57) reported it at 24 months and 36 months. Meta-analysis shown that, compared with its baseline, IBS-QoL score was significantly improved at 1 month, 3 months, 24 months, and 36 months after FMT in the donor stool FMT group (1 month: SMD=0.56, 95%CI [0.29, 0.83]; 3 months: SMD=0.75, 95%CI [0.52, 0.98]; 24 months: MD=27.76, 95%CI [20.79, 34.73]; 36 months: MD=27.61, 95%CI [20.66, 34.56]). However, there were no statistical differences at any time after FMT in the autologous stool FMT group compared with its baseline (1month: SMD=5.10, 95%CI [-3.40, 13.60]; 3 months: SMD=-0.02, 95%CI [-0.52, 0.47]; 24 months: MD=1.80, 95%CI [-8.46, 12.06]; 36 months: MD=-0.60, 95%CI [-9.74, 8.54]).

In both the fecal microbiota capsule FMT group (FMT group) and the placebo capsule FMT group (placebo group), one RCT (46) reported IBS-QoL score at baseline and at 1 month and 3 months after FMT, another RCT (7) reported it at baseline and at 3 months after FMT. Meta-analysis shown that, compared with its baseline, there were no statistical differences at both 1 month and 3 months after FMT in the fecal microbiota capsule FMT group (SMD=-0.49, 95%CI [-1.05, 0.07]; SMD=0.10, 95%CI [-0.30, 0.51]). In the placebo capsule FMT group, the IBS-QoL score was reduced at 1 month after FMT (SMD=-13.73, 95%CI [-22.40, -5.06]), but there was no statistical difference at 3 months after FMT when compared with its baseline (SMD=-0.10, 95%CI [-1.83, 1.63]).

#### Safety of FMT for IBS

The main adverse events reported in these RCTs included abdominal pain, nausea, diarrhea, constipation, bloating or flatulence, headache, fatigue, fever and others. Only one serious adverse event was reported in one RCT (50). A participant in the FMT group was admitted to hospital for a few hours of observation after the FMT procedure due to transient vertigo and nausea, and the researchers deemed this to be related to the medication and instrumentation used during colonoscopy (50). Meta-analysis with random effects model shown that there were no significant statistical differences for these adverse events between the FMT group and placebo group. When I (2) >50%, we removed studies with significant heterogeneity after sensitivity analysis, meta-analysis with fixed effects model shown that FMT may increase the incidence of abdominal pain, constipation, and diarrhea (**Table 3**).

**Table 3:** Meta-analysis results of adverse events.

Adverse events	Reported RCTs	Incidence rate		Heterogeneity		Meta-analysis results (random effects model)		Meta-analysis results (fixed effects model)	
		FMT group	Placebo group	Chi^2^	I^2^	RR	95% CI	RR	95% CI
Any adverse events	6	54.98% (149/271)	36.97% (71/192)	29.71	83%	1.14	0.63, 2.05	0.94#	0.64, 1.38
Abdominal pain	5	14.91% (37/248)	8.43% (14/166)	7.65	48%	1.32	0.51, 3.38	1.96*	1.08, 3.58
Nausea	5	13.30% (33/248)	10.77% (18/167)	1.75	0%	1.22	0.74, 2.03	1.26	0.76, 2.09
Diarrhea	5	19.15% (41/214)	8.02% (13/162)	12.84	69%	2.25	0.56, 9.10	3.81#*	1.28, 11.33
Constipation	3	15.75% (26/165)	1.80% (2/111)	3.87	48%	3.41	0.41, 28.44	5.74*	1.62, 20.32
Bloating/flatulence	4	16.19% (17/105)	14.95% (16/107)	6.8	56%	1.14	0.40, 3.28	1.78#	0.76, 4.19
Headache	3	16.78% (24/143)	6.74 (6/89)	7.75	74%	1.42	0.10, 20.20	0.54#	0.09, 3.34
Fatigue	3	4.87% (4/82)	7.31% (6/82)	1.15	0%	0.68	0.20, 2.30	0.69	0.22, 2.19
Fever	3	3.50% (2/57)	10.16% (6/59)	3.61	45%	0.48	0.05, 4.58	0.48	0.14, 1.67

*The difference was statistically significant between FMT group and placebo group with fixed effects model.

# Fixed effects model was not suitable for meta-analysis because of I^2^>50%, the results were obtained after removing studies with significant heterogeneity.

FMT, fecal microbiota transplantation; RCTs, randomized controlled trials; RR, relative risk; CI, confidence interval.

#### Meta-regression analysis

**Figure 6:**
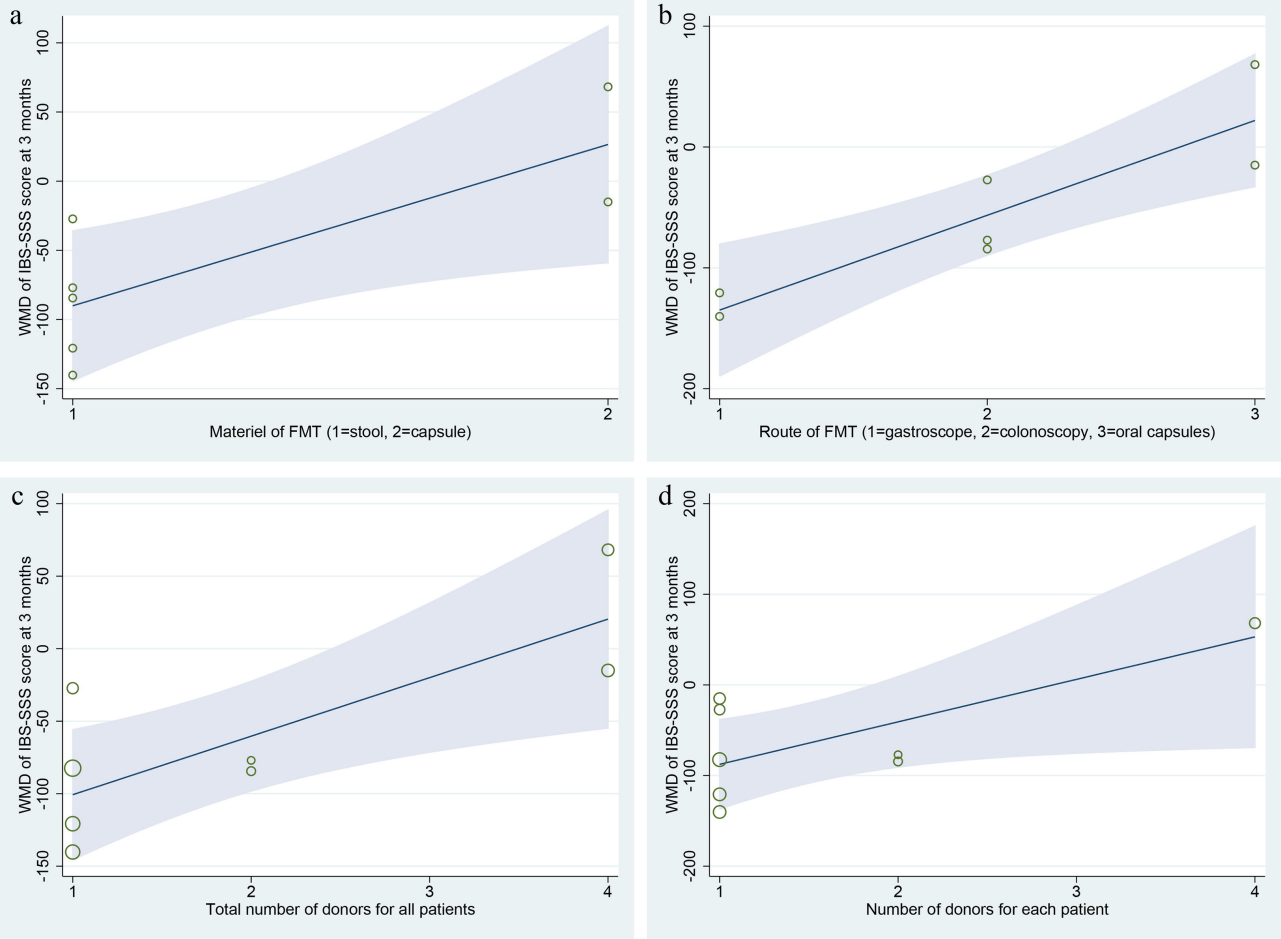
Meta-regression analysis of the primary outcome IBS-SSS score at 3 months after FMT. **(A)** material of FMT (stool vs. capsule); **(B)** route of FMT (gastroscope, colonoscopy, and oral capsules); **(C)** total number of donors for all patients; **(D)** number of donors for each patient (one to one, or mixed to one).

Meta-regression analysis was performed for the primary outcome IBS-SSS score at 3 months after FMT, the covariates included the year of study, material of FMT (stool vs. capsule), route of FMT (gastroscope, colonoscopy, and oral capsules), total number of donors for all patients, number of donors for each patient (one to one, or mixed to one), single dose of stool, total dose of stool, and different style of stool (fresh vs. frozen). The results shown that there were significant relations between IBS-SSS score and these covariates of material of FMT (Coef. = 116.63, p=0.03, 95%CI: 15.23, 218.02), route of FMT (Coef. = 78.45, p=0.00, 95%CI: 34.90, 121.99), total number of donors for all patients (Coef. = 40.36, p=0.01, 95%CI: 9.73, 70.99), and number of donors for each patient (Coef. = 46.87, p=0.04, 95%CI: 0.62, 93.13) (**Figure 6**). However, due to the small number of included RCTs, the statistical reliability of the results above will be significantly reduced, although these four covariates are of great clinical significance.

#### Publication bias

Because of small number of RCTs included, we did not perform a publication bias analysis. However, the included RCTs were mainly small sample studies, and the possibility of publication bias cannot be ruled out (37).

#### Summary of evidences

The summary of findings and the GRADE evidence profile were shown in **Table 4** and **Supplementary Table 4**. In order to comprehensively analyze the effect of FMT on IBS patients, we analyzed the differences between the baseline and endpoint after intervention in different groups, the consistency of different outcomes was shown in **Table 5**, the best consistency of these different outcomes were shown at 3 months, 24 months, and 36 months after stool FMT, and at 3 months after capsule FMT. The risk of bias of study design was shown in **Figure 2**. In summary, the quality was moderate to high in terms of the design of the included studies, and the quality of these primary outcomes after pooled was moderate to low.

**Table 4:** The GRADE evidence profile for the primary outcomes at 3 months/12 weeks.

Quality assessment							No of patients			Effect		Quality	Importance
No	Design	Risk of bias	Inconsistency	Indirectness	Imprecision	Other considerations	FMT	Placebo		Relative (95% CI)/Absolute			
Clinical response rate at 3 months/12 weeks (total)													
5	randomized trials	no serious risk of bias	no serious inconsistency	no serious indirectness	no serious imprecision	reporting bias(1)	172/252 (68.3%)	55/151 (36.4%)		RR 1.6 (0.92 to 2.78)		⊕⊕⊕○ MODERATE	CRITICAL
Subgroup: Clinical response rate at 3 months/12 weeks for stool FMT													
4	randomized trials	no serious risk of bias	no serious inconsistency	no serious indirectness	no serious imprecision	reporting bias(1)	161/230 (70%)	41/128 (32%)		RR 1.91 (1.12 to 3.25)		⊕⊕⊕○ MODERATE	CRITICAL
Subgroup: Clinical response rate at 3 months/12 weeks for capsule FMT													
1	randomized trials	no serious risk of bias	no serious inconsistency	no serious indirectness	serious(2)	reporting bias(1)	11/22 (50%)	14/23 (60.9%)		RR 0.82 (0.48 to 1.4)		⊕⊕⊕○ LOW	CRITICAL
IBS-SSS at 3 months/12 weeks (total)													
6	randomized trials	no serious risk of bias	serious(3)	no serious indirectness	no serious imprecision	reporting bias(1)	247	250		MD -63.77 lower (-103.36 to -24.18 lower)		⊕⊕⊕○ LOW	IMPORTANT
Subgroup: IBS-SSS at 3 months/12 weeks for stool FMT													
3	randomized trials	no serious risk of bias	no serious inconsistency	no serious indirectness	no serious imprecision	reporting bias(1)	188	192		MD -102.11 lower (-141.98 to -62.24 lower)		⊕⊕⊕○ MODERATE	IMPORTANT
Subgroup: IBS-SSS at 3 months/12 weeks for capsule FMT													
3	randomized trials	no serious risk of bias	serious(4)	no serious indirectness	no serious imprecision	reporting bias(1)	59	58		MD -14.19 lower (-103.21 lower to 74.83 higher)		⊕⊕⊕○ LOW	IMPORTANT

^1^ The included studies were all small sample RCTs, which may have significant publication bias. ^2^ The actual sample size was significantly smaller than the optimal information size (OIS). ^3^ Chi^2 = ^40.52, I^2 = ^85%.

^4^ Chi^2 = ^3.52, I^2 = ^72%. FMT, fecal microbiota transplantation; IBS-SSS, irritable bowel syndrome severity scoring system; MD, mean difference; CI, confidence interval.

**Table 5:** Results of this meta-analysis and consistency of different outcomes.

Results	Time	FMT group versus placebo group		FMT group versus its baseline		Placebo group versus its baseline		Consistency of conclusions ^#1^	
		donor stool FMT group versus placebo group	fecal microbiota capsule FMT group versus placebo group	donor stool FMT group versus its baseline	fecal microbiota capsule FMT group versus its baseline	autologous stool FMT group versus its baseline	Placebo capsule FMT group versus its baseline	stool FMT	capsule FMT
**Clinical response rate**	3 months	√↑, 4 RCTs	×, 1 RCT	-	–	-	–	-	–
	6 months	×, 1 RCT	–	-	–	-	–	-	–
	12 months	×, 1 RCT	–	-	–	-	–	-	–
	24 months	√↑, 1 RCT	–	-	–	-	–	-	–
	36 months	√↑, 1 RCT	–	-	–	-	–	-	–
**IBS-SSS**	1 month	√↓, 2 RCTs	×, 2 RCT	√↓, 2 RCTs	√↓, 2 RCT	×, 2 RCTs	×, 2 RCT	Yes	No
	3 months	√↓, 3 RCTs	×*, 3 RCTs	√↓, 2 RCTs	√↓, 3 RCTs	×, 2 RCTs	√↓, 3 RCTs	Yes	Yes
	6 months	√↓, 1 RCTs	×,1 RCT	–	×, 1 RCT	–	√↓, 1 RCT	-	No
	24 months	√↓, 1 RCTs	–	√↓, 1 RCTs	–	×, 1 RCT	–	Yes	–
	36 months	√↓, 1 RCTs	–	√↓, 1 RCTs	–	×, 1 RCT	–	Yes	–
**IBS-QoL**	1 month	×, 1 RCT	×, 1 RCT	√↑, 1 RCT	×, 1 RCT	×, 1 RCT	√↑, 1 RCT	No	No
	3 months	√↑, 2 RCTs	×, 2 RCTs	√↑, 2 RCTs	×, 2 RCTs	×, 2 RCTs	×, 2 RCTs	Yes	Yes
	6 months	-	–	-	–	-	–	-	–
	24 months	√↑, 1 RCT	-	√↑, 1 RCT	-	×, 1 RCT	-	Yes	-
	36 months	√↑, 1 RCT	-	√↑, 1 RCT	-	×, 1 RCT	-	Yes	-
**Abdominal pain**	3 months	√↓, 2 RCTs	×, 1RCTs	–	–	–	–	–	–
**Frequency of stools**	3 months	√↓, 1RCTs	×, 1RCTs	–	–	–	–	–	–
**Stool consistency**	3 months	√↓, 1RCTs	×, 1RCTs	–	–	–	–	–	–
**Consistency of conclusions ^#2^**	1 month	No	Yes	Yes	Yes	Yes	Yes	No	No
	3 months	Yes	Yes	Yes	Yes	Yes	Yes	Yes	Yes
	6 months	-	-	-	-	-	-	-	-
	12 months	-	-	-	-	-	-	-	-
	24 months	Yes	-	Yes	-	Yes	-	Yes	-
	36 months	Yes	-	Yes	-	Yes	-	Yes	-

√, The difference between the two groups compared in the table header was statistically significant; ×, The difference between the two groups compared in the table header was not statistically significant; ↑, The change trend of outcome was upward or increasing; ↓, The change trend of outcome was downward or decreasing. -, The change trend of outcome or the consistency cannot be evaluated.

*For capsule FMT (fecal bacteria capsule vs. placebo capsule), IBS-SSS scores in both the fecal bacteria capsule and placebo capsule groups were significantly lower than their baseline, resulting in no statistical difference in the final outcome between the two groups.

#1, the consistency of the change trend of vertical (FMT group versus placebo group) and horizontal (FMT group/placebo group versus baseline) comparison results of the same outcome at the same time point; #2, the consistency of clinical significance of different outcomes (IBS-SSS score, IBS-QOL score or clinical response rate) at the same time point.

Light green, indicating that the consistency of #1 or #2 in stool FMT group is Yes; Light red, indicating that the consistency of #1 or #2 in capsule FMT group is Yes;

Deep green, indicating that the consistency of both #1 and #2 in stool FMT group is Yes. Deep red, indicating that the consistency of both #1 and #2 in capsule FMT group is Yes.

IBS-SSS, irritable bowel syndrome severity scoring system; IBS-QoL, irritable bowel syndrome specific quality of life; FMT, fecal microbiota transplantation.

## Discussion

As is known, IBS is one of the most common disorders of gut-brain interaction worldwide, its effects on the individual in terms of their quality of life, and on health-care delivery and society in terms of economic costs, are considerable (6, 59). As a non-conventional method, FMT is being explored as a therapeutic option for the patients of IBS. So far, twenty RCTs about FMT for IBS have been registered in the clinicaltrials.gov and International Clinical Trials Registry Platform (**Supplementary Table 5**). Among them, 10 RCTs have been completed, 9 have been included in this study, and the results of one RCT (60) (NCT05088434) was excluded because of imbalanced baseline. Of the 20 registered RCTs, 13 were or would be conducted in European countries, 5 in China, and 2 in the United States, 15 had a sample size of less than 100.

We finally included nine RCTs in this systematic review and meta-analysis, of which five for stool FMT, and four for capsule FMT. Our meta-analysis results shown that the stool FMT could increase the clinical response rate, decrease IBS-SSS score, and improve the quality of life of patients with IBS, without increasing the incidence of serious complications. However, based on the current available data, our study did not confirm the positive effect of capsule FMT on patients with IBS.

The risk of bias of included RCTs was low to moderate. In the stool FMT group, random allocation, allocation concealment and blinding were all performed properly, and the risk of bias was low. In the capsule group, one RCT (54) did not report the specific random allocation scheme, two RCTs (54, 55) did not report whether the allocation concealment for the random sequences was performed, and the risk of bias was moderate (**Figure 2**). In this study, we used the GRADE system to estimate the quality of evidence for these main outcomes, which based on the five factors of risk of bias, inconsistency, indirectness, imprecision, and publication bias. In the stool FMT group, the qualities of the primary outcomes (included clinical response rate and IBS-SSS score) were moderate, which were downgraded by one level due to publication bias, and the reason for publication bias was that all RCTs included were small sample studies. In the capsule FMT group, the qualities of the same primary outcomes were low, which were downgraded by two levels due to publication bias and imprecision, and the reasons for publication bias and inaccuracy were respectively the small sample size of the included studies and the heterogeneity among different studies (**Table 4**). Therefore, it can be seen that the smaller sample size of the included RCTs is one of the main reasons for reducing the reliability of the conclusions for the stool FMT. Predictably, this conclusion will be further confirmed with the emergence of larger RCTs in the future.

As well as stool FMT, capsule FMT has also been shown to be effective and safe in the treatment of recurrent *Clostridioides difficile* infection (13, 61). Halkjær et al. (46) proved that IBS patients in the placebo group experienced greater symptom relief compared with the capsule FMT group after 3 months. Aroniadis et al (7) shown that capsule FMT did not induce symptom relief of IBS patients at 12 weeks compared with placebo. Our study did not also prove that the capsule FMT has a positive therapeutic effect on patients with IBS, but we still refuse to deny the obvious advantages and attractive application prospects of capsule FMT compared with stool FMT. In this systematic review and meta-analysis, the following deficiencies may be the main factors affecting the authenticity of the conclusion that capsule FMT is applied to IBS treatment. First, only four RCTs (7, 46, 54, 55) for capsule FMT were included, the sample size of all included studies was small and the follow-up time was different. As a result, the available data for analysis was insufficient. Second, the heterogeneity among different studies was significant, and the GRADE level of main outcomes was low. Third, in two of the four included studies, the randomization and allocation concealment schemes were unclear, and the risk of bias was moderate. Therefore, it will be very necessary to continue more in-depth and normative research on the application of capsule FMT for patients with IBS in future studies.

Many factors may affect the effectiveness and safety of FMT for patients with IBS (46). These factors were shown in **Tables 1**–**3** in this study. 1) Characteristics of donors. The study by Holvoet et al. (45) shown that higher similarity of microbial community composition between patients and donors at baseline might increase chances of successful FMT in IBS, and the stability of the microbial composition in the donors might be an important predictor of success. Our meta-regression analysis shown that there were significant relations between IBS-SSS score and these covariates of total number of donors for all patients and number of donors for each patient (**Figure 6**). Most of the trials (5/6) that favored FMT used fecal material from one donor, whereas two thirds of the trials that did not favor FMT used mixed fecal material from multiple donors (**Table 2**). The standards for donor screening could refer to the recently published consensus statements for FMT (62–64).

2) Material of FMT. The material styles of FMT included stool and capsule. Of the nine included RCTs, all studies using stool FMT were found to be effective in patients with IBS, while only one of the studies using capsule FMT reached the same conclusion (**Table 2**). Our meta-regression analysis shown that there was significant relation between IBS-SSS score and the material of FMT (**Figure 6**). All fecal microbiota capsule were stored in a frozen state. In this meta-analysis, two RCTs (45, 50) used fresh stool FMT, and four RCTs (20–22, 50) used frozen FMT. Unfortunately, we were unable to perform a subgroup analysis of these two different stool FMT due to the limited data available for extraction. Although frozen stool FMT has been shown to be non-inferior to fresh stool FMT for patients with recurrent *Clostridioides difficile* infection (65, 66), this might not be the case for IBS and warrants further study (7).

3) Route of FMT. Several routes of FMT administration are available at the current, such as nasojejunal tube, gastroscope, duodenoscopy, colonoscopy, enema, and oral capsules. Of which nasojejunal tube (45), gastroscope (21), colonoscopy (20, 22, 50) and oral capsules (7, 46, 54, 55) were used in this study. Meta-regression analysis showed that the route of FMT was correlated with IBS-SSS score at 3 months (**Figure 6**). Similarly, the cure rates of recurrent *Clostridioides difficile* infection with FMT performed with colonoscopy are superior to enema and nasojejunal tube, while FMT with colonoscopy and capsule are comparable (67).

4) Stool dose and frequency of FMT. In the stool FMT group, the single dose of stool was 30g-80g, and in the capsule FMT group, the single dose of stool was 9.5g-50g (**Table 2**). Meta-regression analysis showed that neither single stool dose nor total stool dose of FMT were associated with IBS-SSS score (p>0.05). The frequency of FMT has also differed between included RCTs and might account for differences in results (7). Except for Holvoet et al (45), all other RCTs performed a single FMT administration for the stool FMT in this meta-analysis. Our results shown that, ignoring the different definitions of clinical response rate (**Supplementary Table 3**), the total clinical response rate at 3 months was 70.0% in the stool FMT group and 32.0% in the placebo group. In the study of Holvoet et al (45), the continued response rate was 21% at 1 year after first FMT, and the median time to loss of response was four months (3.5 months-12 months). A second FMT was performed for patients who responded initially to first FMT but lost the effect at 1 year in the study, and it was successful in 67% of patients (45). It suggested that repeated FMT might be a better way to induce a lasting effect in patients with IBS. Previously RCTs that have shown positive results in patients with ulcerative colitis have used an FMT dosing/frequency strategy of enemas once weekly for 6 weeks (68), or 5 days per week for 8 weeks (69, 70).

Four RCTs (7, 46, 54, 55) performed capsule FMT in this systematic review and meta-analysis. The frequencies of oral capsules were respectively 25 capsules per day for 12 days (46), 25 capsules per day for 3 days (7), 19 capsules per day for 1 day (54), and 30 capsule per day for 3 days (55). Three RCTs (7, 46, 54) did not confirm that capsule FMT has a positive effect on patients with IBS, which was consistent with the conclusion of our study. However, considering the effect of dose and frequency of FMT on the results, we suggest that the duration of capsule FMT should be increased to 6-8 weeks in future studies.

5) IBS subtypes. IBS is diagnosed using the Rome criteria, which have volved over the years from the Rome I criteria to the latest Rome IV (71). It is categorized into 4 subtypes based on the predominant stool form or frequency reported by the individual: IBS with constipation (IBS-C); IBS with diarrhea (IBS-D); IBS with mixed bowel habit (IBS-M); or IBS unclassified (IBS-U), where stool form or frequency cannot classify the patient accurately into one of the other 3 subtypes (71). Of the nine RCTs included, eight used Rome III and one used Rome IV, six RCTs included a mixture of patients with differing IBS subtypes, and three RCTs included patients only with IBS-D (**Table 1**). Compared with Rome III, Rome IV is more restrictive and less stable among both functional bowel disorder groups and IBS subtypes (72, 73), the rate of the subtypes change is respectively 24.5% and 31.7% for Rome III and Rome IV in one year (71). Therefore, we are more inclined to support the Rome III as the diagnostic and classification criteria for studies of patients with IBS, and it may be more preferable to subgrouping IBS patients based on the subtypes in future studies.

6) Gender difference. Holvoet et al (45) shown that there was a clear gender difference in the response to FMT, with female patients responding significantly better to active treatment compared to males. However, another RCT (21) found no effect of gender on FMT. Our meta-analysis based on the IBS subtypes shown that the difference in clinical response rate was statistically significant between male and female for patients with IBS-D and IBS-M (two RCTs, RR=0.58, 95% [0.37, 0.89], p=0.01), but there were no differences in IBS-SSS score and IBS-QoL score for IBS patients of all subtypes (P>0.05). It is worth mentioning that this phenomenon exists not only in the FMT process, but also in other IBS treatment options, such as serotonin antagonist alosetron, ibodutant and adding cognitive behavioral therapy to medical treatment, which in favor of effectiveness towards female in either satisfactory relief of overall IBS symptoms or percentage of pain-free days (74). In addition, studies have confirmed that IBS is more common in females (75, 76), and they are more likely to have severe symptoms and coexistent anxiety or depression (77). Thus, gender is one of the possible factors affecting the effect of FMT, which should be paid attention to in future studies.

The safety of FMT for patients with IBS may still one of the focuses of concern (78–80). In this study, seven RCTs (7, 20–22, 46, 50, 54) reported the adverse events, of which most were mild self-limiting gastrointestinal symptoms (**Table 3**). Only one serious adverse event was reported in a participant, who was admitted to hospital for a few hours of observation due to transient vertigo and nausea during colonoscopy (50).

It is generally accepted that IBS is characterized by gut microbiome dysbiosis, but a specific microbial pattern that characterizes all patients with IBS has not been identified due to the lack of consistency in results which seems to be related to the heterogeneity of microbiota assessment (81, 82). All of nine included RCTs reported the characteristics and changes of microbiome profiles after FMT for patients with IBS in this systematic review and meta-analysis, the main conclusions were shown in **Supplementary Table 6**. In all these RCTs, the gut microbiome profiles changed significantly in the groups received FMT. Three RCTs (45, 46, 52) shown that the microbial diversity or richness could be increased after FMT for IBS patients, and five RCTs (7, 20, 22, 46, 52) shown that the microbial composition of the FMT-treated patients shifted towards the donors after the intervention. Holster et al. (53) shown that the microbe-host response was influenced by FMT on the mucosal gene expression level. However, it is a pity that they found none of these changes correlated with clinical improvements. The relationships between the microbiome and the effect of FMT and the etiology of IBS remain unsolved. In addition, microbiota-derived metabolites, such as bile acids, short-chain fatty acids, vitamins, amino acids, serotonin and hypoxanthine, are proposed as possible etiological factors of IBS, and they may provide some new avenues for the diagnosis and treatment of IBS (8, 49).

We believe that this study is the most comprehensive systematic review and meta-analysis so far for use of FMT in patients with IBS. The risk of bias of included RCTs was low in the stool FMT, and was moderate in the capsule FMT. Although there is heterogeneity among different studies, the results of main outcomes obtained after removing the studies with obvious heterogeneity are the same as the former. We used different methods to analyze the quality and reliability of the main outcomes from different perspectives, and the conclusion is reliable (**Figure 2**; **Tables 4**, **5**).

There are some limitations in this RCTs-based meta-analysis, and we put forward some suggestions for future studies about FMT for IBS patients. First, although nine RCTs were included, they were all small sample studies, the qualities of the primary outcomes were downgraded by one level to moderate. In the clinical practice guidelines for IBS published in recent years, FMT was not recommended as a first-line or even second-line treatment because of the low quality of clinical evidence (9, 83, 84). Thus, RCTs with large sample size is urgently needed, which is of great significance to further improve the qualities of outcomes. Second, most of the included RCTs in this meta-analysis were conducted in European countries, and the epidemiological data shown a wide variation in the prevalence of IBS globally. Considering the influence of an individual’s geographical and cultural context on IBS, researches need to be multi­cultural in design, encouraging global collaboration (59). Third, most of the RCTs included a mixture of patients with differing IBS subtypes, and the rate of the subtypes change to each other is significant in one year, it may be more preferable to subgrouping IBS patients based on the subtypes in future studies. Fourth, different outcomes were reported in these RCTs, and different criteria were used to define the same outcomes. We suggest that the clinical remission rate, IBS-SSS Score, IBS-QoL score and other outcomes should be reported in future studies. Clinical remission rate should be defined as IBS-SSS score decreased by ≥50 points after FMT (7, 20, 21, 54), IBS-SSS Score and IBS-QoL score should be measured by using the disease-specific questionnaire (40, 85). Fifth, this meta-analysis showed that a single FMT was effective for IBS patients within 3 months. The median time to loss of response is four months (3.5 months-12 months) (45), and repeated FMT may be a better way to induce a lasting effect in the future studies. Sixth, fecal material from one donor may be better than that from multiple donors in the FMT for a single IBS patient. Seventh, capsule FMT needs to be further studied. Eighth, the relationship between the microbiome and the effect of FMT for IBS is still unclear.

In conclusion, a single stool FMT is effective and safe for patients with IBS, and the efficacy of capsule FMT for IBS remains to be studied in the future. Some factors may affect the effect of FMT, and the relationship between the gut microbiome and FMT for IBS is still unclear.

## Data availability statement

The raw data supporting the conclusions of this article will be made available by the authors, without undue reservation.

## Author contributions

MW and YZ designed the study. MW, XX and YZ independently assessed studies for possible inclusion and collected the data. XX and SZ analyzed the data. MW, ZW, and XM drafted the manuscript. All authors revised and approved the final version of the manuscript. All authors contributed to the article and approved the submitted version.

## Supplementary material

Supplementary Figure 1 Clinical response rate at different times between FMT and placebo groups

Supplementary Figure 2 IBS-SSS score compared FMT and placebo groups at different times

Supplementary Figure 3 IBS-SSS score in the stool FMT group after removed the obviously heterogeneous study

Supplementary Figure 4 IBS-SSS score compared the capsule FMT and placebo groups at different times

Supplementary Figure 5 IBS-QoL score compared FMT and placebo groups at different times

## References

[1] Sperber AD Bangdiwala SI Drossman DA Ghoshal UC Simren M Tack J . Worldwide prevalence and burden of functional gastrointestinal disorders, results of Rome foundation global study. Gastroenterology (2021) 160:99–114.e3. doi: 10.1053/j.gastro.2020.04.014 32294476

[2] Oka P Parr H Barberio B Black CJ Savarino EV Ford AC . Global prevalence of irritable bowel syndrome according to Rome III or IV criteria: a systematic review and meta-analysis. Lancet Gastroenterol Hepatol (2020) 5:908–17. doi: 10.1016/S2468-1253(20)30217-X 32702295

[3] Ford AC Sperber AD Corsetti M Camilleri M . Irritable bowel syndrome. Lancet (2020) 396:1675–88. doi: 10.1016/S0140-6736(20)31548-8 33049223

[4] Flacco ME Manzoli L De Giorgio R Gasbarrini A Cicchetti A Bravi F . Costs of irritable bowel syndrome in European countries with universal healthcare coverage: a meta-analysis. Eur Rev Med Pharmacol Sci (2019) 23:2986–3000. doi: 10.26355/eurrev_201904_17580 31002149

[5] Zhang F Xiang W Li CY Li SC . Economic burden of irritable bowel syndrome in China. World J Gastroenterol (2016) 22:10450–60. doi: 10.3748/wjg.v22.i47.10450 PMC5175258 28058026

[6] Peery AF Crockett SD Murphy CC Jensen ET Kim HP Egberg MD . Burden and cost of gastrointestinal, liver, and pancreatic diseases in the united states: update 2021. Gastroenterology (2022) 162:621–44. doi: 10.1053/j.gastro.2021.10.017 PMC10756322 34678215

[7] Aroniadis OC Brandt LJ Oneto C Feuerstadt P Sherman A Wolkoff AW . Faecal microbiota transplantation for diarrhoea-predominant irritable bowel syndrome: a double-blind, randomised, placebo-controlled trial. Lancet Gastroenterol Hepatol (2019) 4:675–85. doi: 10.1016/S2468-1253(19)30198-0 31326345

[8] Xiao L Liu Q Luo M Xiong L . Gut microbiota-derived metabolites in irritable bowel syndrome. Front Cell Infect Microbiol (2021) 11:729346. doi: 10.3389/fcimb.2021.729346 34631603 PMC8495119

[9] Lacy BE Pimentel M Brenner DM Chey WD Keefer LA Long MD . ACG clinical guideline: management of irritable bowel syndrome. Am J Gastroenterol (2021) 116:17–44. doi: 10.14309/ajg.0000000000001036 33315591

[10] Jeffery IB O'Herlihy E Shanahan F O' Toole PW . Microbiome alterations in IBS. Gut (2020) 69:2263–4. doi: 10.1136/gutjnl-2020-320919 32139549

[11] Liu HN Wu H Chen YZ Chen YJ Shen XZ Liu TT . Altered molecular signature of intestinal microbiota in irritable bowel syndrome patients compared with healthy controls: a systematic review and meta-analysis. Dig Liver Dis (2017) 49:331–7. doi: 10.1016/j.dld.2017.01.142 28179092

[12] Browne PD Cold F Petersen AM Halkjær SI Christensen AH Günther S . Engraftment of strictly anaerobic oxygen-sensitive bacteria in irritable bowel syndrome patients following fecal microbiota transplantation does not improve symptoms. Gut Microbes (2021) 13:1–16. doi: 10.1080/19490976.2021.1927635 PMC8183560 34074214

[13] Hui W Li T Liu W Zhou C Gao F . Fecal microbiota transplantation for treatment of recurrent c. difficile infection: an updated randomized controlled trial meta-analysis. PloS One (2019) 14:e0210016. doi: 10.1371/journal.pone.0210016 30673716 PMC6343888

[14] Hvas CL Dahl Jørgensen SM Jørgensen SP Storgaard M Lemming L Hansen MM . Fecal microbiota transplantation is superior to fidaxomicin for treatment of recurrent clostridium difficile infection. Gastroenterology (2019) 156:1324–1332.e3. doi: 10.1053/j.gastro.2018.12.019 30610862

[15] Caldeira LF Borba HH Tonin FS Wiens A Fernandez-Llimos F Pontarolo R . Fecal microbiota transplantation in inflammatory bowel disease patients: a systematic review and meta-analysis. PloS One (2020) 15:e0238910. doi: 10.1371/journal.pone.0238910 32946509 PMC7500646

[16] El-Salhy M Patcharatrakul T Gonlachanvit S . Fecal microbiota transplantation for irritable bowel syndrome: an intervention for the 21(st) century. World J Gastroenterol (2021) 27:2921–43. doi: 10.3748/wjg.v27.i22.2921 PMC8192290 34168399

[17] Xu D Chen VL Steiner CA Berinstein JA Eswaran S Waljee AK . Efficacy of fecal microbiota transplantation in irritable bowel syndrome: a systematic review and meta-analysis. Am J Gastroenterol (2019) 114:1043–50. doi: 10.14309/ajg.0000000000000198 PMC7257434 30908299

[18] Myneedu K Deoker A Schmulson MJ Bashashati M . Fecal microbiota transplantation in irritable bowel syndrome: a systematic review and meta-analysis. United Eur Gastroenterol J (2019) 7:1033–41. doi: 10.1177/2050640619866990 PMC6794695 31662860

[19] Elhusein AM Fadlalmola HA . Efficacy of fecal microbiota transplantation in irritable bowel syndrome patients: an updated systematic review and meta-analysis. Gastroenterol Nurs (2022) 45:11–20. doi: 10.1097/SGA.0000000000000652 35108241

[20] Lahtinen P Jalanka J Hartikainen A Mattila E Hillilä M Punkkinen J . Randomised clinical trial: faecal microbiota transplantation versus autologous placebo administered via colonoscopy in irritable bowel syndrome. Aliment Pharmacol Ther (2020) 51:1321–31. doi: 10.1111/apt.15740 32343000

[21] El-Salhy M Hatlebakk JG Gilja OH Bråthen Kristoffersen A Hausken T . Efficacy of faecal microbiota transplantation for patients with irritable bowel syndrome in a randomised, double-blind, placebo-controlled study. Gut (2020) 69:859–67. doi: 10.1136/gutjnl-2019-319630 PMC7229896 31852769

[22] Holster S Lindqvist CM Repsilber D Salonen A de Vos WM König J . The effect of allogenic versus autologous fecal microbiota transfer on symptoms, visceral perception and fecal and mucosal microbiota in irritable bowel syndrome: a randomized controlled study. Clin Transl Gastroenterol (2019) 10:e00034. doi: 10.14309/ctg.0000000000000034 31009405 PMC6602784

[23] Higgins JPT TJ Chandler J Cumpston M Li T Page MJ Welch VA . Cochrane handbook for systematic reviews of interventions version 6.2 (2021). Available at: www.training.cochrane.org/handbook. 10.1002/14651858.ED000142 PMC10284251 31643080

[24] Page MJ McKenzie JE Bossuyt PM Boutron I Hoffmann TC Mulrow CD . The PRISMA 2020 statement: an updated guideline for reporting systematic reviews. Bmj (2021) 372:n71. doi: 10.1136/bmj.n71 33782057 PMC8005924

[25] Amir-Behghadami M Janati A . Population, intervention, comparison, outcomes and study (PICOS) design as a framework to formulate eligibility criteria in systematic reviews. Emerg Med J (2020) 37:387. doi: 10.1136/emermed-2020-209567 32253195

[26] Methley AM Campbell S Chew-Graham C McNally R Cheraghi-Sohi S . PICO, PICOS and SPIDER: a comparison study of specificity and sensitivity in three search tools for qualitative systematic reviews. BMC Health Serv Res (2014) 14:579. doi: 10.1186/s12913-014-0579-0 25413154 PMC4310146

[27] Bramer WM Giustini D de Jonge GB Holland L Bekhuis T . De-duplication of database search results for systematic reviews in EndNote. J Med Libr Assoc (2016) 104:240–3. doi: 10.3163/1536-5050.104.3.014 PMC4915647 27366130

[28] Rohatgi A . WebPlotDigitizer 4.5. Pacifica, California, USA (2021). Available at: https://automeris.io/WebPlotDigitizer.

[29] Drevon D Fursa SR Malcolm AL . Intercoder reliability and validity of WebPlotDigitizer in extracting graphed data. Behav Modif (2017) 41:323–39. doi: 10.1177/0145445516673998 27760807

[30] Wan X Wang W Liu J Tong T . Estimating the sample mean and standard deviation from the sample size, median, range and/or interquartile range. BMC Med Res Methodol (2014) 14:135. doi: 10.1186/1471-2288-14-135 25524443 PMC4383202

[31] Shi J Luo D Weng H Zeng X-T Lin L Chu H . Optimally estimating the sample standard deviation from the five-number summary. Res Synth Methods (2020) 11:641–54. doi: 10.1002/jrsm.1429 32562361

[32] Weir CJ Butcher I Assi V Lewis SC Murray GD Langhorne P . Dealing with missing standard deviation and mean values in meta-analysis of continuous outcomes: a systematic review. BMC Med Res Methodol (2018) 18:25. doi: 10.1186/s12874-018-0483-0 29514597 PMC5842611

[33] Guyatt GH Oxman AD Vist G Kunz R Brozek J Alonso-Coello P . GRADE guidelines: 4. rating the quality of evidence–study limitations (risk of bias). J Clin Epidemiol (2011) 64:407–15. doi: 10.1016/j.jclinepi.2010.07.017 21247734

[34] Guyatt GH Oxman AD Kunz R Woodcock J Brozek J Helfand M . GRADE guidelines: 7. rating the quality of evidence–inconsistency. J Clin Epidemiol (2011) 64:1294–302. doi: 10.1016/j.jclinepi.2011.03.017 21803546

[35] Guyatt GH Oxman AD Kunz R Woodcock J Brozek J Helfand M . GRADE guidelines: 8. rating the quality of evidence–indirectness. J Clin Epidemiol (2011) 64:1303–10. doi: 10.1016/j.jclinepi.2011.04.014 21802903

[36] Guyatt GH Oxman AD Kunz R Brozek J Alonso-Coello P Rind D . GRADE guidelines 6. rating the quality of evidence–imprecision. J Clin Epidemiol (2011) 64:1283–93. doi: 10.1016/j.jclinepi.2011.01.012 21839614

[37] Guyatt GH Oxman AD Montori V Vist G Kunz R Brozek J . GRADE guidelines: 5. rating the quality of evidence–publication bias. J Clin Epidemiol (2011) 64:1277–82. doi: 10.1016/j.jclinepi.2011.01.011 21802904

[38] Guyatt G Oxman AD Akl EA Kunz R Vist G Brozek J . GRADE guidelines: 1. introduction-GRADE evidence profiles and summary of findings tables. J Clin Epidemiol (2011) 64:383–94. doi: 10.1016/j.jclinepi.2010.04.026 21195583

[39] Atkins D Best D Briss PA Eccles M Falck-Ytter Y Flottorp S . Grading quality of evidence and strength of recommendations. Bmj (2004) 328:1490. doi: 10.1136/bmj.328.7454.1490 15205295 PMC428525

[40] Francis CY Morris J Whorwell PJ . The irritable bowel severity scoring system: a simple method of monitoring irritable bowel syndrome and its progress. Aliment Pharmacol Ther (1997) 11:395–402. doi: 10.1046/j.1365-2036.1997.142318000.x 9146781

[41] Wiklund IK Fullerton S Hawkey CJ Jones RH Longstreth GF Mayer EA . An irritable bowel syndrome-specific symptom questionnaire: development and validation. Scand J Gastroenterol (2003) 38:947–54. doi: 10.1080/00365520310004209 14531531

[42] Andrade C . Mean difference, standardized mean difference (SMD), and their use in meta-analysis: as simple as it gets. J Clin Psychiatry (2020) 81(5):20f13681. doi: 10.4088/JCP.20f13681 32965803

[43] Higgins JP Thompson SG Deeks JJ Altman DG . Measuring inconsistency in meta-analyses. Bmj (2003) 327:557–60. doi: 10.1136/bmj.327.7414.557 PMC192859 12958120

[44] Sterne JA Sutton AJ Ioannidis JP Terrin N Jones DR Lau J . Recommendations for examining and interpreting funnel plot asymmetry in meta-analyses of randomised controlled trials. Bmj (2011) 343:d4002. doi: 10.1136/bmj.d4002 21784880

[45] Holvoet T Joossens M Vázquez-Castellanos JF Christiaens E Heyerick L Boelens J . Fecal microbiota transplantation reduces symptoms in some patients with irritable bowel syndrome with predominant abdominal bloating: short- and long-term results from a placebo-controlled randomized trial. Gastroenterology (2021) 160:145–157.e8. doi: 10.1053/j.gastro.2020.07.013 32681922

[46] Halkjær SI Christensen AH Lo BZS Browne PD Günther S Hansen LH . Faecal microbiota transplantation alters gut microbiota in patients with irritable bowel syndrome: results from a randomised, double-blind placebo-controlled study. Gut (2018) 67:2107–15. doi: 10.1136/gutjnl-2018-316434 29980607

[47] Madsen AMA Halkjær SI Christensen AH Günther S Browne PD Kallemose T . The effect of faecal microbiota transplantation on abdominal pain, stool frequency, and stool form in patients with moderate-to-severe irritable bowel syndrome: results from a randomised, double-blind, placebo-controlled study. Scand J Gastroenterol (2021) 56:761–9. doi: 10.1080/00365521.2021.1915375 34000958

[48] El-Salhy M Casen C Valeur J Hausken T Hatlebakk JG . Responses to faecal microbiota transplantation in female and male patients with irritable bowel syndrome. World J Gastroenterol (2021) 27:2219–37. doi: 10.3748/wjg.v27.i18.2219 PMC8117742 34025075

[49] El-Salhy M Valeur J Hausken T Gunnar Hatlebakk J . Changes in fecal short-chain fatty acids following fecal microbiota transplantation in patients with irritable bowel syndrome. Neurogastroenterol Motil (2021) 33:e13983. doi: 10.1111/nmo.13983 32945066 PMC7900992

[50] Johnsen PH Hilpüsch F Cavanagh JP Leikanger IS Kolstad C Valle PC . Faecal microbiota transplantation versus placebo for moderate-to-severe irritable bowel syndrome: a double-blind, randomised, placebo-controlled, parallel-group, single-centre trial. Lancet Gastroenterol Hepatol (2018) 3:17–24. doi: 10.1016/S2468-1253(17)30338-2 29100842

[51] Johnsen PH Hilpüsch F Valle PC Goll R . The effect of fecal microbiota transplantation on IBS related quality of life and fatigue in moderate to severe non-constipated irritable bowel: secondary endpoints of a double blind, randomized, placebo-controlled trial. EBioMedicine (2020) 51:102562. doi: 10.1016/j.ebiom.2019.11.023 31877418 PMC6931102

[52] Goll R Johnsen PH Hjerde E Diab J Valle PC Hilpusch F . Effects of fecal microbiota transplantation in subjects with irritable bowel syndrome are mirrored by changes in gut microbiome. Gut Microbes (2020) 12:1794263. doi: 10.1080/19490976.2020.1794263 32991818 PMC7583512

[53] Holster S Hooiveld GJ Repsilber D Vos WM Brummer RJ König J . Allogenic faecal microbiota transfer induces immune-related gene sets in the colon mucosa of patients with irritable bowel syndrome. Biomolecules (2019) 9(10):586. doi: 10.3390/biom9100586 PMC6843426 31597320

[54] Singh P Alm EJ Kelley JM Cheng V Smith M Kassam Z . Effect of antibiotic pretreatment on bacterial engraftment after fecal microbiota transplant (FMT) in IBS-d. Gut Microbes (2022) 14:2020067. doi: 10.1080/19490976.2021.2020067 35014601 PMC8757476

[55] Lin H Guo Q Wen Z Tan S Chen J Lin L . The multiple effects of fecal microbiota transplantation on diarrhea-predominant irritable bowel syndrome (IBS-d) patients with anxiety and depression behaviors. Microb Cell Fact (2021) 20:233. doi: 10.1186/s12934-021-01720-1 34963452 PMC8715582

[56] Mazzawi T Hausken T El-Salhy M . Changes in colonic enteroendocrine cells of patients with irritable bowel syndrome following fecal microbiota transplantation. Scand J Gastroenterol (2022) 57:792–6. doi: 10.1080/00365521.2022.2036809 35156893

[57] El-Salhy M Winkel R Casen C Hausken T Gilja OH Hatlebakk JG . Efficacy of fecal microbiota transplantation for patients with irritable bowel syndrome at 3 years after transplantation. Gastroenterology (2022) 163:982–994.e14. doi: 10.1053/j.gastro.2022.06.020 35709830

[58] El-Salhy M Mazzawi T Hausken T Hatlebakk JG . The fecal microbiota transplantation response differs between patients with severe and moderate irritable bowel symptoms. Scand J Gastroenterol (2022) 57:1036–45. doi: 10.1080/00365521.2022.2064725 35486073

[59] Black CJ Ford AC . Global burden of irritable bowel syndrome: trends, predictions and risk factors. Nat Rev Gastroenterol Hepatol (2020) 17:473–86. doi: 10.1038/s41575-020-0286-8 32296140

[60] Mazzawi T Hausken T Refsnes PF Hatlebakk JG Lied GA . The effect of anaerobically cultivated human intestinal microbiota compared to fecal microbiota transplantation on gut microbiota profile and symptoms of irritable bowel syndrome, a double-blind placebo-controlled study. Microorganisms (2022) 10(9):1819. doi: 10.3390/microorganisms10091819 PMC9503104 36144420

[61] Pomares Bascuñana R Veses V Sheth CC . Effectiveness of fecal microbiota transplant for the treatment of clostridioides difficile diarrhea: a systematic review and meta-analysis. Lett Appl Microbiol (2021) 73:149–58. doi: 10.1111/lam.13486 33864273

[62] Cammarota G Ianiro G Kelly CR Mullish BH Allegretti JR Kassam Z . International consensus conference on stool banking for faecal microbiota transplantation in clinical practice. Gut (2019) 68:2111–21. doi: 10.1136/gutjnl-2019-319548 PMC6872442 31563878

[63] Haifer C Kelly CR Paramsothy S Andresen D Papanicolas LE McKew GL . Australian Consensus statements for the regulation, production and use of faecal microbiota transplantation in clinical practice. Gut (2020) 69:801–10. doi: 10.1136/gutjnl-2019-320260 32047093

[64] Keller JJ Ooijevaar RE Hvas CL Terveer EM Lieberknecht SC Högenauer C . A standardised model for stool banking for faecal microbiota transplantation: a consensus report from a multidisciplinary UEG working group. United Eur Gastroenterol J (2021) 9:229–47. doi: 10.1177/2050640620967898 PMC8259288 33151137

[65] Lee CH Steiner T Petrof EO Smieja M Roscoe D Nematallah A . Frozen vs fresh fecal microbiota transplantation and clinical resolution of diarrhea in patients with recurrent clostridium difficile infection: a randomized clinical trial. Jama (2016) 315:142–9. doi: 10.1001/jama.2015.18098 26757463

[66] Tang G Yin W Liu W . Is frozen fecal microbiota transplantation as effective as fresh fecal microbiota transplantation in patients with recurrent or refractory clostridium difficile infection: a meta-analysis? Diagn Microbiol Infect Dis (2017) 88:322–9. doi: 10.1016/j.diagmicrobio.2017.05.007 28602517

[67] Ramai D Zakhia K Fields PJ Ofosu A Patel G Shahnazarian V . Fecal microbiota transplantation (FMT) with colonoscopy is superior to enema and nasogastric tube while comparable to capsule for the treatment of recurrent clostridioides difficile infection: a systematic review and meta-analysis. Dig Dis Sci (2021) 66:369–80. doi: 10.1007/s10620-020-06185-7 32166622

[68] Moayyedi P Surette MG Kim PT Libertucci J Wolfe M Onischi C . Fecal microbiota transplantation induces remission in patients with active ulcerative colitis in a randomized controlled trial. Gastroenterology (2015) 149:102–109.e6. doi: 10.1053/j.gastro.2015.04.001 25857665

[69] Paramsothy S Kamm MA Kaakoush NO Walsh AJ van den Bogaerde J Samuel D . Multidonor intensive faecal microbiota transplantation for active ulcerative colitis: a randomised placebo-controlled trial. Lancet (2017) 389:1218–28. doi: 10.1016/S0140-6736(17)30182-4 28214091

[70] Paramsothy S Nielsen S Kamm MA Deshpande NP Faith JJ Clemente JC . Specific bacteria and metabolites associated with response to fecal microbiota transplantation in patients with ulcerative colitis. Gastroenterology (2019) 156:1440–1454.e2. doi: 10.1053/j.gastro.2018.12.001 30529583

[71] Barberio B Houghton LA Yiannakou Y Savarino EV Black CJ Ford AC . Symptom stability in Rome IV vs Rome III irritable bowel syndrome. Am J Gastroenterol (2021) 116:362–71. doi: 10.14309/ajg.0000000000000946 33009062

[72] Black CJ Ford AC . Assessing the impact of changes to the Rome IV criteria for clinical practice in irritable bowel syndrome. Gastroenterology (2022) 162(2):1758–4.e1. doi: 10.1053/j.gastro.2022.01.021 35077757

[73] Black CJ Yiannakou Y Houghton LA Ford AC . Epidemiological, clinical, and psychological characteristics of individuals with self-reported irritable bowel syndrome based on the Rome IV vs Rome III criteria. Clin Gastroenterol Hepatol (2020) 18:392–398.e2. doi: 10.1016/j.cgh.2019.05.037 31154027

[74] van Kessel L Teunissen D Lagro-Janssen T . Sex-gender differences in the effectiveness of treatment of irritable bowel syndrome: a systematic review. Int J Gen Med (2021) 14:867–84. doi: 10.2147/IJGM.S291964 PMC7979326 33758534

[75] Chang L Heitkemper MM . Gender differences in irritable bowel syndrome. Gastroenterology (2002) 123:1686–701. doi: 10.1053/gast.2002.36603 12404243

[76] So SY Savidge TC . Sex-bias in irritable bowel syndrome: linking steroids to the gut-brain axis. Front Endocrinol (Lausanne) (2021) 12:684096. doi: 10.3389/fendo.2021.684096 34093447 PMC8170482

[77] Narayanan SP Anderson B Bharucha AE . Sex- and gender-related differences in common functional gastroenterologic disorders. Mayo Clin Proc (2021) 96:1071–89. doi: 10.1016/j.mayocp.2020.10.004 PMC8075061 33814075

[78] Janket SJ Ackerson LK Diamandis EP . Drug-resistant bacteremia after fecal microbiota transplant. N Engl J Med (2020) 382:1960. doi: 10.1056/NEJMc2002496 32402173

[79] DeFilipp Z Bloom PP Torres Soto M Mansour MK Sater MRA Huntley MH . Drug-resistant e. coli bacteremia transmitted by fecal microbiota transplant. N Engl J Med (2019) 381:2043–50. doi: 10.1056/NEJMoa1910437 31665575

[80] Blaser MJ . Fecal microbiota transplantation for dysbiosis - predictable risks. N Engl J Med (2019) 381:2064–6. doi: 10.1056/NEJMe1913807 31665573

[81] Pittayanon R Lau JT Yuan Y Leontiadis GI Tse F Surette M . Gut microbiota in patients with irritable bowel syndrome-a systematic review. Gastroenterology (2019) 157:97–108. doi: 10.1053/j.gastro.2019.03.049 30940523

[82] Wang L Alammar N Singh R Nanavati J Song Y Chaudhary R . Gut microbial dysbiosis in the irritable bowel syndrome: a systematic review and meta-analysis of case-control studies. J Acad Nutr Diet (2020) 120:565–86. doi: 10.1016/j.jand.2019.05.015 31473156

[83] Vasant DH Paine PA Black CJ Houghton LA Everitt HA Corsetti M . British Society of gastroenterology guidelines on the management of irritable bowel syndrome. Gut (2021) 70:1214–40. doi: 10.1136/gutjnl-2021-324598 33903147

[84] Smalley W Falck-Ytter C Carrasco-Labra A Wani S Lytvyn L Falck-Ytter Y . AGA clinical practice guidelines on the laboratory evaluation of functional diarrhea and diarrhea-predominant irritable bowel syndrome in adults (IBS-d). Gastroenterology (2019) 157:851–4. doi: 10.1053/j.gastro.2019.07.004 31302098

[85] Drossman DA Patrick DL Whitehead WE Toner BB Diamant NE Hu Y . Further validation of the IBS-QOL: a disease-specific quality-of-life questionnaire. Am J Gastroenterol (2000) 95:999–1007. doi: 10.1111/j.1572-0241.2000.01941.x 10763950

