# Supplementary material for: Fecal microbiota transplantation for irritable bowel syndrome: a systematic review and meta-analysis of randomized controlled trials
Source: Front Immunol. 2023 May 18;14:1136343. doi: 10.3389/fimmu.2023.1136343 (PMC10234428; doi:10.3389/fimmu.2023.1136343)
Supplement: Supplementary Figure 1 — Clinical response rate at different times between FMT and placebo groups [file DataSheet_1.zip › Supplementary materials/Supplementary table 1-The full search syntaxes for databases.docx]

Supplementary table 1. The full search syntaxes for databases

| **Search number** | **Query** |
| --- | --- |
| #1 | (fecal) OR (fecal[MeSH Terms]) |
| #2 | (faecal) OR (faecal[MeSH Terms]) |
| #3 | (feces) OR (feces[MeSH Terms]) |
| #4 | (faeces) OR (faeces[MeSH Terms]) |
| #5 | (stool) OR (stool[MeSH Terms]) |
| #6 | #1 OR #2 OR #3 OR #4 OR #5 |
| #7 | (microbiota) OR (microbiota[MeSH Terms]) |
| #8 | (microbiome) OR (microbiome[MeSH Terms]) |
| #9 | (bacteria) OR (bacteria[MeSH Terms]) |
| #10 | (microflora) OR (microflora[MeSH Terms]) |
| #11 | #7 OR #8 OR #9 OR #10 |
| #12 | (transplantation) OR (transplantation[MeSH Terms]) |
| #13 | (transplant) OR (transplant[MeSH Terms]) |
| #14 | (transfer) OR (transfer[MeSH Terms]) |
| #15 | #12 OR #13 OR #14 |
| #16 | #6 AND #11 AND #15 |
| #17 | (irritable bowel syndrome) OR (irritable bowel syndrome[MeSH Terms]) |
| #18 | (IBS) OR (IBS[MeSH Terms]) |
| #19 | #17 OR #18 |
| #20 | #16 AND #19, Filters: Randomized Controlled Trial/Clinical Trial |
