# Supplementary material for: Fecal microbiota transplantation for irritable bowel syndrome: a systematic review and meta-analysis of randomized controlled trials
Source: Front Immunol. 2023 May 18;14:1136343. doi: 10.3389/fimmu.2023.1136343 (PMC10234428; doi:10.3389/fimmu.2023.1136343)
Supplement: Supplementary Figure 1 — Clinical response rate at different times between FMT and placebo groups [file DataSheet_1.zip › Supplementary materials/Supplementary table 1.pdf]

Supplementary table 1. The full search syntaxes for databases

**Search number**	**Query**
#1	(fecal) OR (fecal[MeSH Terms])
#2	(faecal) OR (faecal[MeSH Terms])
#3	(feces) OR (feces[MeSH Terms])
#4	(faeces) OR (faeces[MeSH Terms])
#5	(stool) OR (stool[MeSH Terms])
#6	#1 OR #2 OR #3 OR #4 OR #5
#7	(microbiota) OR (microbiota[MeSH Terms])
#8	(microbiome) OR (microbiome[MeSH Terms])
#9	(bacteria) OR (bacteria[MeSH Terms])
#10	(microflora) OR (microflora[MeSH Terms])
#11	#7 OR #8 OR #9 OR #10
#12	(transplantation) OR (transplantation[MeSH Terms])
#13	(transplant) OR (transplant[MeSH Terms])
#14	(transfer) OR (transfer[MeSH Terms])
#15	#12 OR #13 OR #14
#16	#6 AND #11 AND #15
#17	(irritable bowel syndrome) OR (irritable bowel syndrome[MeSH Terms])
#18	(IBS) OR (IBS[MeSH Terms])
#19	#17 OR #18
#20	#16 AND #19, Filters: Randomized Controlled Trial/Clinical Trial
