# Supplementary material for: Fecal microbiota transplantation for irritable bowel syndrome: a systematic review and meta-analysis of randomized controlled trials
Source: Front Immunol. 2023 May 18;14:1136343. doi: 10.3389/fimmu.2023.1136343 (PMC10234428; doi:10.3389/fimmu.2023.1136343)
Supplement: Supplementary Figure 1 — Clinical response rate at different times between FMT and placebo groups [file DataSheet_1.zip › Supplementary materials/Supplementary table 2-The main outcomes and design of included studies.docx]

Supplementary table 2. The main outcomes and design of included studies

| **Trial ID** | **Author, Year** | **Outcomes** | **Design of trial** |
| --- | --- | --- | --- |
| NCT02299973 | Holvoet T, 2021 | response rate*, IBS symptoms*, bloating*, IBS-symptom-scores, IBS-QoL*, microbiome profiles*, sex difference* | single-center, double-blinded, cross-over randomized controlled trial. |
| NCT02788071 | Halkjær SI, 2018 | IBS-SSS, IBS-QoL, Bristol Stool Form Scale, symptoms, use of laxatives, side effects*, microbiome profiles | double-center, double-blinded, randomized controlled trial. |
|  | Madsen AMA, 2021 | abdominal pain, stool frequency, stool form |  |
|  | Browne PD, 2021 | α-diversity, β-diversity, microbiome profiles* |  |
| NCT03822299 | El-Salhy M, 2020 | response rate, IBS-SSS, Birmingham IBS-S, Fatigue (FAS), IBS- QoL, SF-NDI, dysbiosis index, microbiome profiles*, side effects | single-center, double- blinded, randomized controlled trial. |
|  | El-Salhy M, 2021 | sex difference in the response to FMT |  |
|  | El-Salhy M, 2021 | short-chain fatty acids* |  |
| NCT03561519 | Lahtinen P, 2020 | response rate, IBS-SSS*, IBS-QoL, depression (BDI), anxiety (BAI), general quality of life (15D), microbiome profiles*, side effects | multi-center, double-blinded, randomized controlled trial. |
| NCT02154867 | Johnsen PH, 2018 | response rate at 3 months* and 12 months, IBS-SSS, side effects | single-center, double-blinded, parallel-group, randomized controlled trial. |
|  | Johnsen PH, 2020 | fatigue*, IBS-QoL*, effect of diet, other functional disorders, mood disorders (anxiety and depression) |  |
|  | Goll R, 2020 | α-diversity*, β-diversity* |  |
| NCT02328547 | Aroniadis OC, 2019 | IBS-SSS, IBS-QoL, response rate, depression, anxiety, stool consistency, microbiome profiles*, side effects | multi-center, double-blinded, cross-over randomized controlled trial. |
| NCT02092402 | Holster S, 2019 | GSRS-IBS, IBS-SSS, IBS-QoL, response rate, depression, anxiety, SF-36, Visceral sensitivity, microbiome profiles*, side effects | single-center, double- blinded, randomized controlled trial. |
|  | Holster S, 2019 | Immune-related gene sets in the colon mucosa* |  |
| NCT02847481 | Singh P, 2022 | Change in IBS-SSS, Change in IBS-QoL, adequate relief, global improvement, response rate, side effects, engraftment rate*，microbiome profiles | single-center, double-blinded, randomized controlled trial. |
| ChiCTR1900024924 | Lin H, 2021 | IBS-SSS*, IBS-QoL*, depression*, anxiety*, microbiome profiles*, short-chain fatty acids* | single-center, double-blinded, randomized controlled trial. |

*The difference was statistically significant between FMT and placebo groups.

IBS-SSS, irritable bowel syndrome severity scoring system; IBS-QoL, irritable bowel syndrome specific quality of life; FMT, fecal microbiota transplantation; GSRS, gastrointestinal symptom rating scale; SF-NDI, short- form nepean dyspepsia index; HAD: hospital anxiety depression.
