# Supplementary material for: Fecal microbiota transplantation for irritable bowel syndrome: a systematic review and meta-analysis of randomized controlled trials
Source: Front Immunol. 2023 May 18;14:1136343. doi: 10.3389/fimmu.2023.1136343 (PMC10234428; doi:10.3389/fimmu.2023.1136343)
Supplement: Supplementary Figure 1 — Clinical response rate at different times between FMT and placebo groups [file DataSheet_1.zip › Supplementary materials/Supplementary table 2.pdf]

Supplementary table 2. The main outcomes and design of included studies

Trial ID	Author, Year	Outcomes	Design of trial
NCT02299973	Holvoet T, 2021	response rate*, IBS symptoms*, bloating*, IBS-symptom-scores, IBS-QoL*, microbiome profiles*, sex difference*	single-center, double-blinded, cross-over randomized controlled trial.
NCT02788071	Halkjær SI, 2018	IBS-SSS, IBS-QoL, Bristol Stool Form Scale, symptoms, use of laxatives, side effects*, microbiome profiles	double-center, double-blinded, randomized controlled trial.
	Madsen AMA, 2021	abdominal pain, stool frequency, stool form	
	Browne PD, 2021	α-diversity, β-diversity, microbiome profiles*	
NCT03822299	El-Salhy M, 2020	response rate, IBS-SSS, Birmingham IBS-S, Fatigue (FAS), IBS- QoL, SF-NDI, dysbiosis index, microbiome profiles*, side effects	single-center, double- blinded, randomized controlled trial.
	El-Salhy M, 2021	sex difference in the response to FMT	
	El-Salhy M, 2021	short-chain fatty acids*	
NCT03561519	Lahtinen P, 2020	response rate, IBS-SSS*, IBS-QoL, depression (BDI), anxiety (BAI), general quality of life (15D), microbiome profiles*, side effects	multi-center, double-blinded, randomized controlled trial.
NCT02154867	Johnsen PH, 2018	response rate at 3 months* and 12 months, IBS-SSS, side effects	single-center, double-blinded, parallel-group, randomized controlled trial.
	Johnsen PH, 2020	fatigue*, IBS-QoL*, effect of diet, other functional disorders, mood disorders (anxiety and depression)	
	Goll R, 2020	α-diversity*, β-diversity*	
NCT02328547	Aroniadis OC, 2019	IBS-SSS, IBS-QoL, response rate, depression, anxiety, stool consistency, microbiome profiles*, side effects	multi-center, double-blinded, cross-over randomized controlled trial.
NCT02092402	Holster S, 2019	GSRS-IBS, IBS-SSS, IBS-QoL, response rate, depression, anxiety, SF-36, Visceral sensitivity, microbiome profiles*, side effects	single-center, double- blinded, randomized controlled trial.
	Holster S, 2019	Immune-related gene sets in the colon mucosa*	
NCT02847481	Singh P, 2022	Change in IBS-SSS, Change in IBS-QoL, adequate relief, global improvement, response rate, side effects, engraftment rate*, microbiome profiles	single-center, double-blinded, randomized controlled trial.

ChiCTR1900024924	Lin H, 2021	IBS-SSS*, IBS-QoL*, depression*, anxiety*, microbiome profiles*, short-chain fatty acids*	single-center, double-blinded, randomized controlled trial.

*The difference was statistically significant between FMT and placebo groups.

IBS-SSS, irritable bowel syndrome severity scoring system; IBS-QoL, irritable bowel syndrome specific quality of life; FMT, fecal microbiota transplantation; GSRS, gastrointestinal symptom rating scale; SF-NDI, short- form nepean dyspepsia index.
