# Supplementary material for: Fecal microbiota transplantation for irritable bowel syndrome: a systematic review and meta-analysis of randomized controlled trials
Source: Front Immunol. 2023 May 18;14:1136343. doi: 10.3389/fimmu.2023.1136343 (PMC10234428; doi:10.3389/fimmu.2023.1136343)
Supplement: Supplementary Figure 1 — Clinical response rate at different times between FMT and placebo groups [file DataSheet_1.zip › Supplementary materials/Supplementary table 3-The definition of clinical response rate in different studies.docx]

Supplementary table 3. The definition of clinical response rate in different studies

| **Trial ID** | **Definition of clinical response rate** | **Style of FMT** |
| --- | --- | --- |
| NCT02299973 | Self-reported adequate relief of general IBS symptoms based on a daily symptom diary evaluation | stool |
| NCT03822299 | IBS-SSS score decreased by ≥ 50 | stool |
| NCT03561519 | IBS-SSS score decreased by ≥ 50 | stool |
| NCT02154867 | IBS-SSS score decreased by ≥ 75 | stool |
| NCT02328547 | IBS-SSS score decreased by ≥ 50 | capsule |
| NCT02092402 | GSRS-IBS symptom score decrease by ≥ 30% | stool |
| NCT02847481 | IBS-SSS score decreased by ≥ 50 | capsule |

NCT, national clinical trial; FMT, fecal microbiota transplantation.

* NCT02847481 reported the clinical response rate at 10 weeks after FMT, NCT02092402 reported it at 6 months, and others at 3 months.
