# Supplementary material for: Fecal microbiota transplantation for irritable bowel syndrome: a systematic review and meta-analysis of randomized controlled trials
Source: Front Immunol. 2023 May 18;14:1136343. doi: 10.3389/fimmu.2023.1136343 (PMC10234428; doi:10.3389/fimmu.2023.1136343)
Supplement: Supplementary Figure 1 — Clinical response rate at different times between FMT and placebo groups [file DataSheet_1.zip › Supplementary materials/Supplementary table 3.pdf]

Supplementary table 3. The definition of clinical response rate in different studies

**Trial ID**	**Definition of clinical response rate**	**Style of FMT**
NCT02299973	Self-reported adequate relief of general IBS symptoms based on a daily symptom diary evaluation	stool
NCT03822299	IBS-SSS score decreased by ≥ 50	stool
NCT03561519	IBS-SSS score decreased by ≥ 50	stool
NCT02154867	IBS-SSS score decreased by ≥ 75	stool
NCT02328547	IBS-SSS score decreased by ≥ 50	capsule
NCT02092402	GSRS-IBS symptom score decrease by ≥ 30%	stool
NCT02847481	IBS-SSS score decreased by ≥ 50	capsule

NCT, national clinical trial; FMT, fecal microbiota transplantation.

* NCT02847481 reported the clinical response rate at 10 weeks after FMT, NCT02092402 reported it at 6 months, and others at 3 months.
