# Supplementary material for: Fecal microbiota transplantation for irritable bowel syndrome: a systematic review and meta-analysis of randomized controlled trials
Source: Front Immunol. 2023 May 18;14:1136343. doi: 10.3389/fimmu.2023.1136343 (PMC10234428; doi:10.3389/fimmu.2023.1136343)
Supplement: Supplementary Figure 1 — Clinical response rate at different times between FMT and placebo groups [file DataSheet_1.zip › Supplementary materials/Supplementary table 4-The summary of findings and the GRADE evidence profile for other outcomes at different time points.docx]

Supplementary table 4. The summary of findings and the GRADE evidence profile for other outcomes at different time points

| **Quality assessment** | | | | | | | **No of patients** | | **Effect** | | **Quality** | **Importance** |
| --- | --- | --- | --- | --- | --- | --- | --- | --- | --- | --- | --- | --- |
| **No of studies** | **Design** | **Risk of bias** | **Inconsistency** | **Indirectness** | **Imprecision** | **Other considerations** | **FMT** | **Placebo** | **Relative (95% CI)** | **Absolute** |  |  |
| **Clinical response rate for stool FMT at 6 months** | | | | | | | | | | | | |
| 1 | randomised trials | no serious risk of bias | no serious inconsistency | no serious indirectness | serious^2^ | reporting bias^1^ | 4/8  (50%) | 1/8  (12.5%) | RR 4  (0.56 to 28.4) | 375 more per 1000 (from 55 fewer to 1000 more) | ⊕⊕OO LOW | NOT IMPORTANT |
| **Clinical response rate for stool FMT at 12 months** | | | | | | | | | | | | |
| 1 | randomised trials | no serious risk of bias | no serious inconsistency | no serious indirectness | serious^2^ | reporting bias^1^ | 31/55  (56.4%) | 10/28  (35.7%) | RR 1.58 (0.91 to 2.73) | 207 more per 1000 (from 32 fewer to 618 more) | ⊕⊕OO LOW | NOT IMPORTANT |
| **IBS-SSS at 1 months/ 4 weeks (total)** | | | | | | | | | | | | |
| 4 | randomised trials | no serious risk of bias | serious^3^ | no serious indirectness | serious^2^ | reporting bias^1^ | 167 | 171 | - | MD -55.72 lower (-105.01 to -6.43 lower) | ⊕⊕OO LOW | NOT IMPORTANT |
| **Subgroup: IBS-SSS at 1 months/ 4 weeks for stool FMT** | | | | | | | | | | | | |
| 3 | randomised trials | no serious risk of bias | no serious inconsistency | no serious indirectness | serious^2^ | reporting bias^1^ | 133 | 136 | - | MD -65.75 lower (-129.37 to -2.13 lower) | ⊕⊕OO LOW | NOT IMPORTANT |
| **Subgroup: IBS-SSS at 1 months/ 4 weeks for capsule FMT** | | | | | | | | | | | | |
| 1 | randomised trials | no serious risk of bias | no serious inconsistency | no serious indirectness | serious^2^ | reporting bias^1^ | 34 | 35 | - | MD -33.11 lower (-180.69 lower to 114.47 higher) | ⊕⊕OO LOW | NOT IMPORTANT |
| **IBS-SSS at 6 months (total)** | | | | | | | | | | | | |
| 2 | randomised trials | no serious risk of bias | no serious inconsistency | no serious indirectness | serious^2^ | reporting bias^1^ | 80 | 82 | - | MD -27.87 lower (-138.28 lower to 82.54 higher) | ⊕⊕OO LOW | IMPORTANT |
| **Subgroup: IBS-SSS at 6 months for stool FMT** | | | | | | | | | | | | |
| 1 | randomised trials | no serious risk of bias | no serious inconsistency | no serious indirectness | serious^2^ | reporting bias^1^ | 55 | 56 | - | MD -84.38 lower (-158.79 to -9.97 lower) | ⊕⊕OO LOW | IMPORTANT |
| **Subgroup: IBS-SSS at 6 months for capsule FMT** | | | | | | | | | | | | |
| 1 | randomised trials | no serious risk of bias | no serious inconsistency | no serious indirectness | serious^2^ | reporting bias^1^ | 25 | 26 | - | MD 67.03 higher (-3.53 lower to 137.59 higher) | ⊕⊕OO LOW | IMPORTANT |
| **IBS-SSS core for stool FMT at 52 weeks** | | | | | | | | | | | | |
| 1 | randomised trials | no serious risk of bias | no serious inconsistency | no serious indirectness | serious^2^ | reporting bias^1^ | 23 | 26 | - | MD -12.68 lower (-82.76 lower to 57.4 higher) | ⊕⊕OO LOW | NOT IMPORTANT |
| **IBS-QoL at 1 months (total)** | | | | | | | | | | | | |
| 2 | randomised trials | no serious risk of bias | no serious inconsistency | no serious indirectness | serious^2^ | reporting bias^1^ | 135 | 136 | - | SMD 0.14 higher (-0.11 lower to 0.38 higher) | ⊕⊕OO LOW | NOT IMPORTANT |
| **Subgroup: IBS-QoL at 1 months for stool FMT** | | | | | | | | | | | | |
| 1 | randomised trials | no serious risk of bias | no serious inconsistency | no serious indirectness | serious^2^ | reporting bias^1^ | 110 | 110 | - | SMD 0.07 higher (-0.2 lower to 0.33 higher) | ⊕⊕OO LOW | NOT IMPORTANT |
| **Subgroup: IBS-QoL at 1 months for capsule FMT** | | | | | | | | | | | | |
| 1 | randomised trials | no serious risk of bias | no serious inconsistency | no serious indirectness | serious^2^ | reporting bias^2^ | 25 | 26 | - | SMD 0.43 higher (-0.12 lower to 0.99 higher) | ⊕⊕OO LOW | NOT IMPORTANT |
| **IBS-QoL at 3 months (total)** | | | | | | | | | | | | |
| 4 | randomised trials | no serious risk of bias | no serious inconsistency | no serious indirectness | serious^2^ | reporting bias^1^ | 203 | 178 | - | SMD 0.62 higher (0.33 to 0.9 higher) | ⊕⊕OO LOW | NOT IMPORTANT |
| **Subgroup: IBS-QoL at 3 months for stool FMT** | | | | | | | | | | | | |
| 2 | randomised trials | no serious risk of bias | no serious inconsistency | no serious indirectness | no serious imprecision^2^ | reporting bias^1^ | 153 | 129 | - | SMD 0.78 higher (0.53 to 1.02 higher) | ⊕⊕⊕O MODERATE | NOT IMPORTANT |
| **Subgroup: IBS-QoL at 3 months for capsules FMT** | | | | | | | | | | | | |
| 2 | randomised trials | no serious risk of bias | serious^6^ | no serious indirectness | serious^2^ | reporting bias^2^ | 50 | 49 | - | SMD 0.3 higher (-0.4 lower to 1 higher) | ⊕⊕OO LOW | NOT IMPORTANT |
| **Abdominal pain at 3 months (total)** | | | | | | | | | | | | |
| 3 | randomised trials | no serious risk of bias | serious^7^ | no serious indirectness | no serious imprecision | reporting bias^1^ | 178 | 155 | - | SMD -0.38 lower (-0.8 lower to 0.04 higher) | ⊕⊕OO LOW | NOT IMPORTANT |
| **Subgroup: Abdominal pain at 3 months for stool FMT** | | | | | | | | | | | | |
| 2 | randomised trials | no serious risk of bias | no serious inconsistency | no serious indirectness | no serious imprecision | reporting bias^1^ | 153 | 129 | - | SMD -0.6 lower (-0.84 to -0.35 lower) | ⊕⊕⊕O MODERATE | NOT IMPORTANT |
| **Subgroup: Abdominal pain at 3 months for capsules FMT** | | | | | | | | | | | | |
| 1 | randomised trials | no serious risk of bias | no serious inconsistency | no serious indirectness | serious^2^ | reporting bias^2^ | 25 | 26 | - | SMD 0.38 higher (-0.17 lower to 0.93 higher) | ⊕⊕OO LOW | NOT IMPORTANT |
| **Frequency of stools at 3 months (total)** | | | | | | | | | | | | |
| 2 | randomised trials | no serious risk of bias | no serious inconsistency | no serious indirectness | serious^2^ | reporting bias^2^ | 68 | 45 | - | MD -0.34 lower (-0.7 lower to 0.02 higher) | ⊕⊕OO LOW | NOT IMPORTANT |
| **Subgroup: Frequency of stools at 3 months for stool FMT** | | | | | | | | | | | | |
| 1 | randomised trials | no serious risk of bias | no serious inconsistency | no serious indirectness | serious^2^ | reporting bias^2^ | 43 | 19 | - | MD -0.5 lower (-0.93 to -0.07 lower) | ⊕⊕OO LOW | NOT IMPORTANT |
| **Subgroup: Frequency of stools at 3 months for capsules FMT** | | | | | | | | | | | | |
| 1 | randomised trials | no serious risk of bias | no serious inconsistency | no serious indirectness | serious^2^ | reporting bias^1^ | 25 | 26 | - | MD 0.02 higher (-0.63 lower to 0.67 higher) | ⊕⊕OO LOW | NOT IMPORTANT |
| **Stool consistency at 3 months (total)** | | | | | | | | | | | | |
| 2 | randomised trials | no serious risk of bias | serious^8^ | no serious indirectness | serious^2^ | reporting bias^1^ | 68 | 45 | - | MD -0.19 lower (-0.42 lower to 0.04 higher) | ⊕⊕OO LOW | NOT IMPORTANT |
| **Subgroup: Stool consistency at 3 months for stool FMT** | | | | | | | | | | | | |
| 1 | randomised trials | no serious risk of bias | no serious inconsistency | no serious indirectness | serious^2^ | reporting bias^1^ | 43 | 19 | - | MD -0.33 lower (-0.61 to -0.05 lower) | ⊕⊕OO LOW | NOT IMPORTANT |
| **Subgroup: Stool consistency at 3 months for capsule FMT** | | | | | | | | | | | | |
| 1 | randomised trials | no serious risk of bias | no serious inconsistency | no serious indirectness | serious^2^ | reporting bias^1^ | 25 | 26 | - | MD 0.06 higher (-0.32 lower to 0.44 higher) | ⊕⊕OO LOW | NOT IMPORTANT |

^1^ The included studies were all small sample RCTs, which may have significant publication bias. ^2^ The actual sample size was significantly smaller than the optimal information size (OIS). ^3^ Chi^2^=25.10, I^2^=88%. ^4^ Chi^2^=40.52, I^2^=85%. ^5^ Chi^2^=3.52, I^2^=72%. ^6^ Chi^2^=3.05, I^2^=67%. ^7^ Chi^2^=10.05, I^2^=70%. ^8^ Chi^2^=2.56, I^2^=61%.

FMT, fecal microbiota transplantation; IBS-SSS, irritable bowel syndrome severity scoring system; QoL, quality of life. RR, relative risk; MD, mean difference; CI, confidence interval.
