# Supplementary material for: Fecal microbiota transplantation for irritable bowel syndrome: a systematic review and meta-analysis of randomized controlled trials
Source: Front Immunol. 2023 May 18;14:1136343. doi: 10.3389/fimmu.2023.1136343 (PMC10234428; doi:10.3389/fimmu.2023.1136343)
Supplement: Supplementary Figure 1 — Clinical response rate at different times between FMT and placebo groups [file DataSheet_1.zip › Supplementary materials/Supplementary table 4.pdf]

Supplementary table 4. The summary of findings and the GRADE evidence profile for other outcomes at different time points

**Quality assessment**							**No of patients**		**Effect**		**Quality**	**Importance**
**No of studies**	**Design**	**Risk of bias**	**Inconsistency**	**Indirectness**	**Imprecision**	**Other considerations**	**FMT**	**Placebo**	**Relative (95% CI)**	**Absolute**		
**Clinical response rate for stool FMT at 6 months**												
1	randomised trials	no serious risk of bias	no serious inconsistency	no serious indirectness	serious[2]	reporting bias[1]	4/8 (50%)	1/8 (12.5%)	RR 4 (0.56 to 28.4)	375 more per 1000 (from 55 fewer to 1000 more)	⊕⊕OO LOW	NOT IMPORTANT
**Clinical response rate for stool FMT at 12 months**												
1	randomised trials	no serious risk of bias	no serious inconsistency	no serious indirectness	serious[2]	reporting bias[1]	31/55 (56.4%)	10/28 (35.7%)	RR 1.58 (0.91 to 2.73)	207 more per 1000 (from 32 fewer to 618 more)	⊕⊕OO LOW	NOT IMPORTANT
**IBS-SSS at 1 months/ 4 weeks (total)**												
4	randomised trials	no serious risk of bias	serious[3]	no serious indirectness	serious[2]	reporting bias[1]	167	171	-	MD -55.72 lower (-105.01 to -6.43 lower)	⊕⊕OO LOW	NOT IMPORTANT
**Subgroup: IBS-SSS at 1 months/ 4 weeks for stool FMT**												
3	randomised trials	no serious risk of bias	no serious inconsistency	no serious indirectness	serious[2]	reporting bias[1]	133	136	-	MD -65.75 lower (-129.37 to -2.13 lower)	⊕⊕OO LOW	NOT IMPORTANT
**Subgroup: IBS-SSS at 1 months/ 4 weeks for capsule FMT**												
1	randomised trials	no serious risk of bias	no serious inconsistency	no serious indirectness	serious[2]	reporting bias[1]	34	35	-	MD -33.11 lower (-180.69 lower to 114.47 higher)	⊕⊕OO LOW	NOT IMPORTANT
**IBS-SSS at 6 months (total)**												
2	randomised trials	no serious risk of bias	no serious inconsistency	no serious indirectness	serious[2]	reporting bias[1]	80	82	-	MD -27.87 lower (-138.28 lower to 82.54 higher)	⊕⊕OO LOW	IMPORTANT
**Subgroup: IBS-SSS at 6 months for stool FMT**												
1	randomised	no serious	no serious	no serious	serious[2]	reporting bias[1]	55	56	-	MD -84.38 lower (-158.79	⊕⊕OO	IMPORTANT

	trials	risk of bias	inconsistency	indirectness						to -9.97 lower)	LOW	
**Subgroup: IBS-SSS at 6 months for capsule FMT**												
1	randomised trials	no serious risk of bias	no serious inconsistency	no serious indirectness	serious[2]	reporting bias[1]	25	26	-	MD 67.03 higher (-3.53 lower to 137.59 higher)	⊕⊕OO LOW	IMPORTANT
**IBS-SSS core for stool FMT at 52 weeks**												
1	randomised trials	no serious risk of bias	no serious inconsistency	no serious indirectness	serious[2]	reporting bias[1]	23	26	-	MD -12.68 lower (-82.76 lower to 57.4 higher)	⊕⊕OO LOW	NOT IMPORTANT
**IBS-QoL at 1 months (total)**												
2	randomised trials	no serious risk of bias	no serious inconsistency	no serious indirectness	serious[2]	reporting bias[1]	135	136	-	SMD 0.14 higher (-0.11 lower to 0.38 higher)	⊕⊕OO LOW	NOT IMPORTANT
**Subgroup: IBS-QoL at 1 months for stool FMT**												
1	randomised trials	no serious risk of bias	no serious inconsistency	no serious indirectness	serious[2]	reporting bias[1]	110	110	-	SMD 0.07 higher (-0.2 lower to 0.33 higher)	⊕⊕OO LOW	NOT IMPORTANT
**Subgroup: IBS-QoL at 1 months for capsule FMT**												
1	randomised trials	no serious risk of bias	no serious inconsistency	no serious indirectness	serious[2]	reporting bias[2]	25	26	-	SMD 0.43 higher (-0.12 lower to 0.99 higher)	⊕⊕OO LOW	NOT IMPORTANT
**IBS-QoL at 3 months (total)**												
4	randomised trials	no serious risk of bias	no serious inconsistency	no serious indirectness	serious[2]	reporting bias[1]	203	178	-	SMD 0.62 higher (0.33 to 0.9 higher)	⊕⊕OO LOW	NOT IMPORTANT
**Subgroup: IBS-QoL at 3 months for stool FMT**												
2	randomised trials	no serious risk of bias	no serious inconsistency	no serious indirectness	no serious imprecision[2]	reporting bias[1]	153	129	-	SMD 0.78 higher (0.53 to 1.02 higher)	⊕⊕⊕O MODERATE	NOT IMPORTANT
**Subgroup: IBS-QoL at 3 months for capsules FMT**												
2	randomised	no serious	serious[6]	no serious	serious[2]	reporting bias[2]	50	49	-	SMD 0.3 higher (-0.4 lower	⊕⊕OO	NOT

	trials	risk of bias		indirectness						to 1 higher)	LOW	IMPORTANT
**Abdominal pain at 3 months (total)**												
3	randomised trials	no serious risk of bias	serious[7]	no serious indirectness	no serious imprecision	reporting bias[1]	178	155	-	SMD -0.38 lower (-0.8 lower to 0.04 higher)	⊕⊕OO LOW	NOT IMPORTANT
**Subgroup: Abdominal pain at 3 months for stool FMT**												
2	randomised trials	no serious risk of bias	no serious inconsistency	no serious indirectness	no serious imprecision	reporting bias[1]	153	129	-	SMD -0.6 lower (-0.84 to -0.35 lower)	⊕⊕⊕O MODERATE	NOT IMPORTANT
**Subgroup: Abdominal pain at 3 months for capsules FMT**												
1	randomised trials	no serious risk of bias	no serious inconsistency	no serious indirectness	serious[2]	reporting bias[2]	25	26	-	SMD 0.38 higher (-0.17 lower to 0.93 higher)	⊕⊕OO LOW	NOT IMPORTANT
**Frequency of stools at 3 months (total)**												
2	randomised trials	no serious risk of bias	no serious inconsistency	no serious indirectness	serious[2]	reporting bias[2]	68	45	-	MD -0.34 lower (-0.7 lower to 0.02 higher)	⊕⊕OO LOW	NOT IMPORTANT
**Subgroup: Frequency of stools at 3 months for stool FMT**												
1	randomised trials	no serious risk of bias	no serious inconsistency	no serious indirectness	serious[2]	reporting bias[2]	43	19	-	MD -0.5 lower (-0.93 to -0.07 lower)	⊕⊕OO LOW	NOT IMPORTANT
**Subgroup: Frequency of stools at 3 months for capsules FMT**												
1	randomised trials	no serious risk of bias	no serious inconsistency	no serious indirectness	serious[2]	reporting bias[1]	25	26	-	MD 0.02 higher (-0.63 lower to 0.67 higher)	⊕⊕OO LOW	NOT IMPORTANT
**Stool consistency at 3 months (total)**												
2	randomised trials	no serious risk of bias	serious[8]	no serious indirectness	serious[2]	reporting bias[1]	68	45	-	MD -0.19 lower (-0.42 lower to 0.04 higher)	⊕⊕OO LOW	NOT IMPORTANT
**Subgroup: Stool consistency at 3 months for stool FMT**												
1	randomised	no serious	no serious	no serious	serious[2]	reporting bias[1]	43	19	-	MD -0.33 lower (-0.61 to -	⊕⊕OO	NOT

	trials	risk of bias	inconsistency	indirectness						0.05 lower)	LOW	IMPORTANT
**Subgroup: Stool consistency at 3 months for capsule FMT**												
1	randomised trials	no serious risk of bias	no serious inconsistency	no serious indirectness	serious[2]	reporting bias[1]	25	26	-	MD 0.06 higher (-0.32 lower to 0.44 higher)	⊕⊕OO LOW	NOT IMPORTANT

[1] The included studies were all small sample RCTs, which may have significant publication bias. [2] The actual sample size was significantly smaller than the optimal information size (OIS). [3] Chi^2=25.10, I^2=88%. [4] Chi^2=40.52, I^2=85%. [5] Chi^2=3.52, I^2=72%. [6] Chi^2=3.05, I^2=67%. [7] Chi^2=10.05, I^2=70%. [8] Chi^2=2.56, I^2=61%.

FMT, fecal microbiota transplantation; IBS-SSS, irritable bowel syndrome severity scoring system; QoL, quality of life. RR, relative risk; MD, mean difference; CI, confidence interval.
