# Supplementary material for: Fecal microbiota transplantation for irritable bowel syndrome: a systematic review and meta-analysis of randomized controlled trials
Source: Front Immunol. 2023 May 18;14:1136343. doi: 10.3389/fimmu.2023.1136343 (PMC10234428; doi:10.3389/fimmu.2023.1136343)
Supplement: Supplementary Figure 1 — Clinical response rate at different times between FMT and placebo groups [file DataSheet_1.zip › Supplementary materials/Supplementary table 5-.docx]

Supplementary table 5. RCTs about FMT for IBS that have been registered in the clinicaltrials.gov and ICTRP

| **No.** | **Trial ID** | **Title** | **Interventions** | **RCT** | **Estimated Enrollment** | **Study Start Date** | **Status** | **Study Results** | **Locations** |
| --- | --- | --- | --- | --- | --- | --- | --- | --- | --- |
| 1 | **NCT02299973** | Fecal Microbiota Transplantation in Irritable Bowel Syndrome With Bloating | FMT with donor stool FMT with autologous stool | Yes | 64 participants | October 2014 | Completed | Available | Ghent University Hospital, Ghent, Belgium |
| 2 | **NCT02092402** | Fecal Microbiota Transplantation in Patients With Irritable Bowel Syndrome | FMT with donor stool FMT with autologous stool | Yes | 17 participants | September 2013 | Completed | Available | Robert Brummer, Örebro University, Sweden |
| 3 | **NCT02328547** | Fecal Microbiota Transplantation for the Treatment of Diarrhea-Predominant Irritable Bowel Syndrome | FMT capsules Placebo capsules | Yes | 48 participants | May 2015 | Completed | Available | Medical Research Center of Connecticut, Hamden, Connecticut, United States |
| 4 | NCT04691544 | Donor Versus Autologous Fecal Microbiota Transplantation for Irritable Bowel Syndrome | FMT with donor stool FMT with autologous stool | Yes | 450 participants | May 5, 2021 | Recruiting | No available | University Hospital of North Norway |
| 5 | NCT03613545 | Fecal Microbiota Transplantation for Irritable Bowel Syndrome | FMT with donor stool FMT with autologous stool | Yes | 120 participants | August 10, 2018 | Recruiting | No available | Guangzhou First People's Hospital, Guangzhou, Guangdong, China |
| 6 | NCT03074227 | The FAIS-Trial: Faecal Microbiota Transplantation (FMT) in Adolescents With Refractory Irritable Bowel Syndrome (IBS) (FAIS) | FMT with donor stool FMT with autologous stool | Yes | 30 participants | November 23, 2017 | Recruiting | No available | AMC, Amsterdam, Noord Holland, Netherlands |
| 7 | **NCT02788071** | Effect of Fecal Microbiota Transplantation in Irritable Bowel Syndrome | FMT capsules Placebo capsules | Yes | 52 participants | October 2016 | Completed | Available | Aleris Hamlet Hospitaler, København , Copenhagen, Denmark |
| 8 | **NCT02847481** | A Study to Evaluate Fecal Microbiota Transplantation Engraftment in IBS | FMT capsules Placebo capsules | Yes | 80 participants | May 2016 | Completed | Available | Beth Israel Deaconess Medical Center, Boston, Massachusetts, United States |
| 9 | NCT04890405 | Clinical Study of Selective Fecal Microbiota Transplantation in the Treatment of Irritable Bowel Syndrome. | Standardized FMT capsules Precision transplant capsules | Yes | 70 participants | May 20, 2021 | Not recruiting | No available | Army Medical Center of PLA,Chongqing, China |
| 10 | NCT02423421 | Faecal Microbiota Transplantation in Irritable Bowel Syndrome | FMT with donor stool FMT with autologous stool | Yes | 50 participants | March 2015 | Unknown | No available | Alimentary Pharmabiotic Centre, University College Cork, Cork, Ireland |
| 11 | NCT03125564 | FMT for Patients With IBS With Fecal and Mucosal Microbiota Assessment | FMT with donor stool Infusion of sham | Yes | 56 participants | April 12, 2017 | Not recruiting | No available | The Chinese University of Hong Kong, Hong Kong, China |
| 12 | NCT04899869 | Faecal Microbiota Transplantation in Irritable Bowel Syndrome (MISCEAT) | FMT with donor stool Placebo by autoclaving | Yes | 100 participants | June 17, 2021 | Recruiting | No available | Thomayer University Hospital, Prague, Czechia |
| 13 | **NCT02154867** | Fecal Microbial Transplantation in Treatment of Irritable Bowel Syndrome; a Double Blinded Placebo Controlled Trial. (REFIT) | FMT with donor stool FMT with autologous stool | Yes | 90 participants | December 2014 | Completed | Available | University Hospital of North Norway, Harstad, Norway |
| 14 | NCT05088434 | Fecal Microbiota Transplantation and ACHIM for Manipulating Gut Microbiota in IBS Patients | FMT with donor stool Anaerobically cultivated human intestinal microbiota FMT with autologous stool | Yes | 62 participants | January 1, 2017 | Completed | No available | Haukeland Unversity Hospital, Bergen, Norway |
| 15 | **NCT03561519** | FMT in the Treatment of IBS (FMT-IBS) | FMT with donor stool FMT with autologous stool | Yes | 52 participants | August 27, 2015 | Completed | Available | Helsinki University Hospital, Helsinki, Finland |
| 16 | NCT04236843 | Faecal Microbiota Transplantation (FMT) in Patients With IBSmechanism(s) of Action | FMT with donor stool (into small intestine once, twice, and into large intestine once) | Yes | 186 participants | January 1, 2020 | Not recruiting | No available | Helse Fonna, Haugesund, Norway |
| 17 | **NCT03822299** | Effects of Faecal Microbiota Transplantation in Patients With IBS | FMT with donor stool FMT with autologous stool | Yes | 165 participants | January 1, 2018 | Completed | Available | Helse Fonna, Haugesund, Norway |
| 18 | **ChiCTR1900024924** | A clinical study for the effect of the fecal microbiota transplantation on the treatment of the irritable bowel syndrome combined with anxiety or depression | FMT capsules Placebo capsules | Yes | 18 participants | August 1, 2019 | Completed | Available | PingXiang People's Hospital, Jiangxi, China |
| 19 | ChiCTR-INR- 17013457 | Efficacy and safety of Intestinal bacteria capsule for the Treatment of refractory Diarrhea-Predominant Irritable Bowel Syndrome | FMT capsules Probiotics | Yes | 50 participants | November 20, 2017 | Unknown | No available | The Affiliated Third Hospital of Army Medical University, Chongqing, China |
| 20 | DRKS00010941 | Fecal microbiota transplantation in irritable bowel syndrome: a double blind, placebo controlled trial. | FMT with donor stool FMT with autologous stool | Yes | 72 participants | March 16, 2016 | Unknown | No available | Klinische Abteilung für Gastroenterologie Medizinische Universität Graz, Graz, Austria |
