# Supplementary material for: Fecal microbiota transplantation for irritable bowel syndrome: a systematic review and meta-analysis of randomized controlled trials
Source: Front Immunol. 2023 May 18;14:1136343. doi: 10.3389/fimmu.2023.1136343 (PMC10234428; doi:10.3389/fimmu.2023.1136343)
Supplement: Supplementary Figure 1 — Clinical response rate at different times between FMT and placebo groups [file DataSheet_1.zip › Supplementary materials/Supplementary table 5.pdf]

Supplementary table 5. RCTs about FMT for IBS that have been registered in the clinicaltrials.gov and ICTRP

**No.**	**Trial ID**	**Title**	**Interventions**	**RCT**	**Estimated Enrollment**	**Study Start Date**	**Status**	**Study Results**	**Locations**
1	**NCT02299973**	Fecal Microbiota Transplantation in Irritable Bowel Syndrome With Bloating	FMT with donor stool<br>FMT with autologous stool	Yes	64 participants	October 2014	Completed	Available	Ghent University Hospital, Ghent, Belgium
2	**NCT02092402**	Fecal Microbiota Transplantation in Patients With Irritable Bowel Syndrome	FMT with donor stool<br>FMT with autologous stool	Yes	17 participants	September 2013	Completed	Available	Robert Brummer, Örebro University, Sweden
3	**NCT02328547**	Fecal Microbiota Transplantation for the Treatment of Diarrhea-Predominant Irritable Bowel Syndrome	FMT capsules<br>Placebo capsules	Yes	48 participants	May 2015	Completed	Available	Medical Research Center of Connecticut, Hamden, Connecticut, United States
4	NCT04691544	Donor Versus Autologous Fecal Microbiota Transplantation for Irritable Bowel Syndrome	FMT with donor stool<br>FMT with autologous stool	Yes	450 participants	May 5, 2021	Recruiting	No available	University Hospital of North Norway
5	NCT03613545	Fecal Microbiota Transplantation for Irritable Bowel Syndrome	FMT with donor stool<br>FMT with autologous stool	Yes	120 participants	August 10, 2018	Recruiting	No available	Guangzhou First People's Hospital, Guangzhou, Guangdong, China
6	NCT03074227	The FAIS-Trial: Faecal Microbiota Transplantation (FMT) in Adolescents With Refractory Irritable Bowel Syndrome (IBS) (FAIS)	FMT with donor stool<br>FMT with autologous stool	Yes	30 participants	November 23, 2017	Recruiting	No available	AMC, Amsterdam, Noord Holland, Netherlands
7	**NCT02788071**	Effect of Fecal Microbiota Transplantation in Irritable Bowel Syndrome	FMT capsules<br>Placebo capsules	Yes	52 participants	October 2016	Completed	Available	Aleris Hamlet Hospitaler, København , Copenhagen, Denmark
8	**NCT02847481**	A Study to Evaluate Fecal Microbiota Transplantation Engraftment in IBS	FMT capsules<br>Placebo capsules	Yes	80 participants	May 2016	Completed	Available	Beth Israel Deaconess Medical Center, Boston, Massachusetts, United States

9	NCT04890405	Clinical Study of Selective Fecal Microbiota Transplantation in the Treatment of Irritable Bowel Syndrome.	Standardized FMT capsules<br>Precision transplant capsules	Yes	70 participants	May 20, 2021	Not recruiting	No available	Army Medical Center of PLA,Chongqing, China
10	NCT02423421	Faecal Microbiota Transplantation in Irritable Bowel Syndrome	FMT with donor stool<br>FMT with autologous stool	Yes	50 participants	March 2015	Unknown	No available	Alimentary Pharmabiotic Centre, University College Cork, Cork, Ireland
11	NCT03125564	FMT for Patients With IBS With Fecal and Mucosal Microbiota Assessment	FMT with donor stool<br>Infusion of sham	Yes	56 participants	April 12, 2017	Not recruiting	No available	The Chinese University of Hong Kong, Hong Kong, China
12	NCT04899869	Faecal Microbiota Transplantation in Irritable Bowel Syndrome (MISCEAT)	FMT with donor stool<br>Placebo by autoclaving	Yes	100 participants	June 17, 2021	Recruiting	No available	Thomayer University Hospital, Prague, Czechia
13	**NCT02154867**	Fecal Microbial Transplantation in Treatment of Irritable Bowel Syndrome; a Double Blinded Placebo Controlled Trial. (REFIT)	FMT with donor stool<br>FMT with autologous stool	Yes	90 participants	December 2014	Completed	Available	University Hospital of North Norway, Harstad, Norway
14	NCT05088434	Fecal Microbiota Transplantation and ACHIM for Manipulating Gut Microbiota in IBS Patients	FMT with donor stool<br>Anaerobically cultivated human intestinal microbiota<br>FMT with autologous stool	Yes	62 participants	January 1, 2017	Completed	No available	Haukeland Unversity Hospital, Bergen, Norway
15	**NCT03561519**	FMT in the Treatment of IBS (FMT-IBS)	FMT with donor stool<br>FMT with autologous stool	Yes	52 participants	August 27, 2015	Completed	Available	Helsinki University Hospital, Helsinki, Finland
16	NCT04236843	Faecal Microbiota Transplantation (FMT)<br>in Patients With IBSmechanism(s) of Action	FMT with donor stool (into small intestine once, twice, and into large intestine once)	Yes	186 participants	January 1, 2020	Not recruiting	No available	Helse Fonna, Haugesund, Norway

17	**NCT03822299**	Effects of Faecal Microbiota Transplantation in Patients With IBS	FMT with donor stool<br>FMT with autologous stool	Yes	165 participants	January 1, 2018	Completed	Available	Helse Fonna, Haugesund, Norway
18	**ChiCTR1900024924**	A clinical study for the effect of the fecal microbiota transplantation on the treatment of the irritable bowel syndrome combined with anxiety or depression	FMT capsules<br>Placebo capsules	Yes	18 participants	August 1, 2019	Completed	Available	PingXiang People's Hospital, Jiangxi, China
19	ChiCTR-INR-17013457	Efficacy and safety of Intestinal bacteria capsule for the Treatment of refractory Diarrhea-Predominant Irritable Bowel Syndrome	FMT capsules<br>Probiotics	Yes	50 participants	November 20, 2017	Unknown	No available	The Affiliated Third Hospital of Army Medical University, Chongqing, China
20	DRKS00010941	Fecal microbiota transplantation in irritable bowel syndrome: a double blind, placebo controlled trial.	FMT with donor stool<br>FMT with autologous stool	Yes	72 participants	March 16, 2016	Unknown	No available	Klinische Abteilung für Gastroenterologie Medizinische Universität Graz, Graz, Austria
