# Supplementary material for: Fecal microbiota transplantation for irritable bowel syndrome: a systematic review and meta-analysis of randomized controlled trials
Source: Front Immunol. 2023 May 18;14:1136343. doi: 10.3389/fimmu.2023.1136343 (PMC10234428; doi:10.3389/fimmu.2023.1136343)
Supplement: Supplementary Figure 1 — Clinical response rate at different times between FMT and placebo groups [file DataSheet_1.zip › Supplementary materials/Supplementary table 6.pdf]

Supplementary table 6. The characteristics and changes of microbiome profiles in the included RCTs after FMT for patients with IBS

Number	Trial ID	FMT material	Microbiome assessment method	Characteristics and changes of microbiome profiles after FMT
1	NCT02299973	stool	16S rRNA genes V4 region	Higher similarity between patient and donor at baseline might increase chances of successful FMT in IBS, and the stability of the microbial composition in the donors might be an important predictor of success.<br>IBS patients that responded to the FMT treatment had a higher microbial diversity and overall bacterial composition at baseline as compared to those who failed active FMT treatment, so response associated with composition of the fecal microbiomes before FMT.<br>We could not identify taxa that were predictive of success in our cohort.
2	NCT02788071	capsule	16S rRNA genes V3-V4 regions	Patients receiving FMT capsules had an increase in biodiversity to the extent that this group was not statistically distinguishable from the donors.<br>FMT-treated patients' microbiota became more similar to the donors', both short- and long-term. A larger proportion of the FMT groups' microbiotas originated from the FMT donors' microbiotas in contrast with the placebo group. Major long-term changes, lasting for at least 6 months, in the gut microbiomes of IBS patients are seen following treatment with FMT capsules.<br>There were no correlations between IBS-SSS and α-diversity. FMT-treated patients having significantly more of the OTU than the patients at inclusion and the placebo patients,none of these OTUs showed any significant changes in abundance due to FMT treatment.<br>FMT induces both significant engraftment and loss of bacteria. Lost much higher numbers of ASVs than the placebo patients. Increasing in abundance in the FMT group relative to the placebo group include Faecalibacterium, Bacteroides, Prevotella and Lactobacillus.<br>None of these changes correlated with clinical improvement.
3	NCT03822299	stool	16S rRNA gene V3-V7 regions	Faecal bacterial profiles showed significant changes in the abundance of bacteria in the 30 g FMT and 60 g FMT groups but not in the placebo group.<br>The intestinal bacterial profiles changed significantly in the groups received FMT. Alistipes spp., Bacte-roides, Prevotella spp., Firmicutes spp., Eubacterium biforme, Lactobacillus spp. and Akkermansia muciniphilawere increased for FMT, while Bacteroides spp., Dorea spp. and Eubacterium hallii decreased.<br>The IBS-SSS score was significantly correlated with the concentrations of Lactobacillus spp. and Alistipes spp., but not with that of Bacteroides spp. or Eubacterium biforme.

4	NCT03561519	stool	16S rRNA gene V3-V4 regions	The microbial richness in both groups was lower than that of the donor, the microbial richness increased significantly only in the FMT group. However, the increase in microbial richness was not reflected as an increase in the microbial diversity.<br>The microbial composition of the FMT-treated patients shifted towards the donor after the intervention, whereas there was no significant difference in the placebo group.
5	NCT02154867	stool	Metagenomics analysis by an Illumina NextSeq550 instrument with a NextSeq 500/550 High output v2 kit	Thirty-four functional subclasses showed distinct differences between baseline samples and donors, most of which were shifted toward a donor-like profile after FMT. FMT induces long-term changes in gut microbiota, and these changes mirror the clinical effect of the treatment.<br>The richness (Chao1 and Observed) was significantly higher in the Effect group than in the Donors at all time points for baseline, 6 months, and 12 months.<br>The microbial composition of fecal samples from the Effect group clustered closer to the Donor samples after 12 month, the No effect samples did not show any separation in clustering between the sampling time points.<br>The Donor samples had a relatively higher abundance of Bacteroidetes than the patient samples, and lower abundance of Firmicutes and Actinobacteria. The relative abundance profiles changed over the sampling period in both the Effect and No effect groups, becoming more similar to the Donor profile with increased abundance Bacteroidetes and a reduction of Firmicutes. For the No effect group, however, the 6 month samples, in particular, had relatively low abundance of Bacteroides and relatively high abundance of Actinobacteria.<br>In total, 170 species showed a significantly different abundance in response to FMT, of these, 128 species increased in abundance and 42 decreased in abundance after FMT.<br>Twenty-four of the 36 species belonging to the Firmicutes phylum showed a reduced abundance following FMT. In phylum Bacteroidetes, 64 of 67 species increased in abundance after FMT. Of species known to be involved in the production of short-chain fatty acids (SCFA), 3 of 4 Eubacterium sp., 0 of 2 Clostridium sp., 11 of 12 Ruminococcus sp., 2 of 2 Klebsiella sp., and 2 of 2 Lactobacillus sp. had a decreased abundance after FMT. However, considering baseline counts of these species, the resulting SCFA production capacity increased by a factor 6.7 after FMT.
6	NCT02092402	capsule	16S rRNA gene V4 region	Community diversity, quantified by the Shannon diversity index, among FMT responders was not different from that of non-responders at baseline or weeks 1, 4, or 12.<br>The Jensen-Shannon distance between the patient and donor microbiomes decreased between the timepoint before FMT and1week after FMT, suggesting that the bacterial communities of the FMT recipients became more similar to that of the donors.<br>A supervised machine learning classifier did not successfully predict FMT response with statistical significance from bacterial taxonomic features. Bacterial communities of some FMT responders had high ratios of Bacteroidetes to Firmicutes

				and a high abundance of Prevotella at baseline.
7	NCT02092402	stool	Human Intestinal Tract Chip	Fecal samples from the allogenic group 2 and 8 weeks after FMT seemed to cluster closer to their corresponding donor, especially for recipients of fecal material from donor 1. The mucosal microbiota of the recipients did not seem to cluster closely to the mucosal microbiota of the donors.<br>The patients with a positive symptom response did not show increased fecal or mucosal microbiota diversity.
8	NCT02847481	capsule	16S rRNA gene V4 region	No striking differences in microbial composition were noted 1-week and 10-week post-FMT/placebo vs. baseline in both groups;<br>Antibiotic pre-treatment significantly reduced the bacterial engraftment after FMT suggesting that future FMT trials in IBS should not be preceded by antibiotics.
9	ChiCTR1900024924	capsule	Illumina HiSeq4000 platform	At the genus level, the abundance of dominant genera, such as Bacteroides and Phocaeicola, were significantly increased by FMT treatment. Besides, the level of Bifidobacterium was also showed a significant increasing trend in the FMT group. Conversely, FMT treatment decreased the abundance of Faecalibacterium, Eubacterium and Escherichia.
