# Supplementary figures and images for: Fecal microbiota transplantation for irritable bowel syndrome: a systematic review and meta-analysis of randomized controlled trials
Source: Front Immunol. 2023 May 18;14:1136343. doi: 10.3389/fimmu.2023.1136343 (PMC10234428; doi:10.3389/fimmu.2023.1136343)

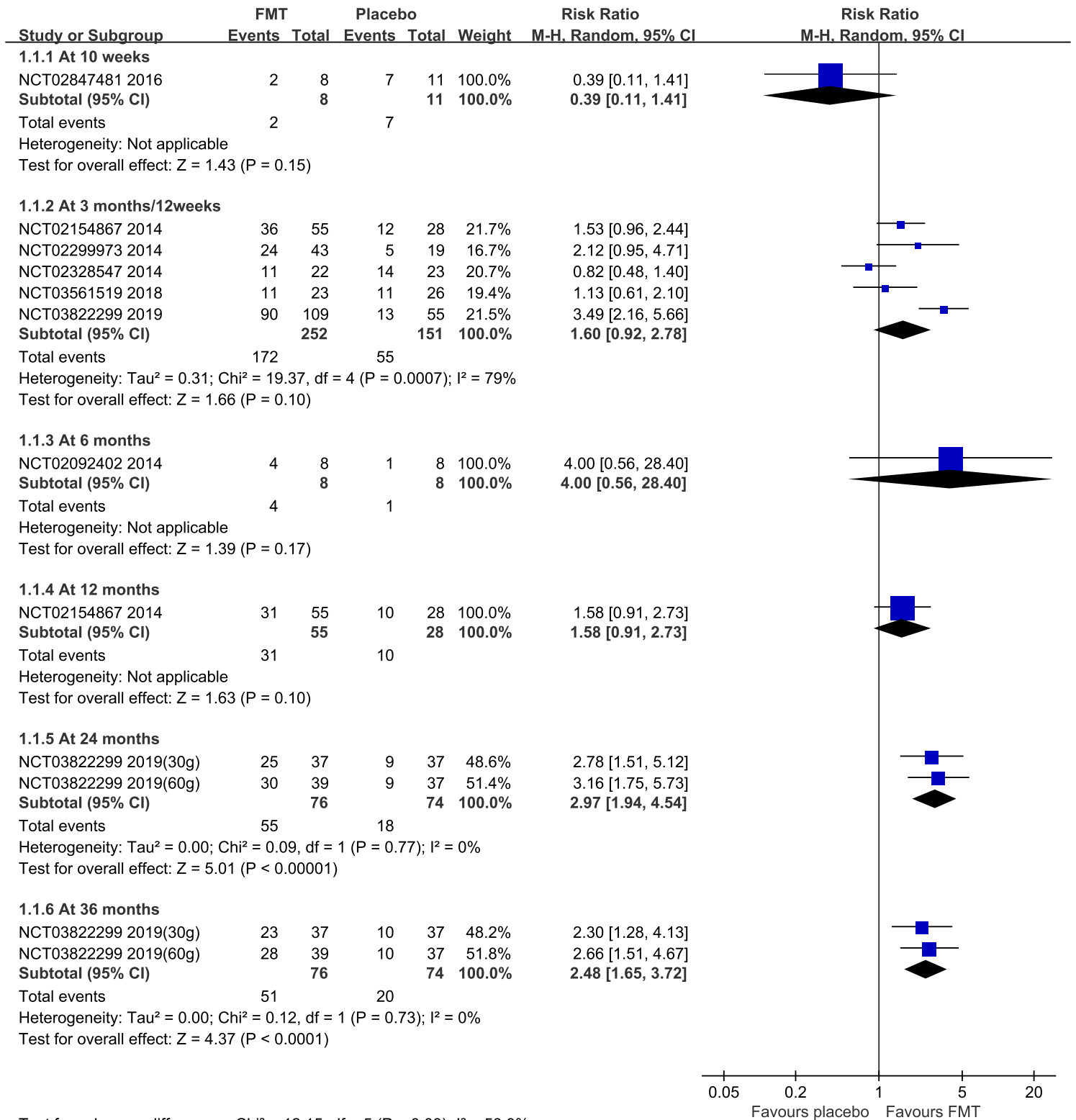

Supplement: Supplementary Figure 1 — Clinical response rate at different times between FMT and placebo groups [file DataSheet_1.zip › Supplementary materials/Supplementary figure 1.pdf]

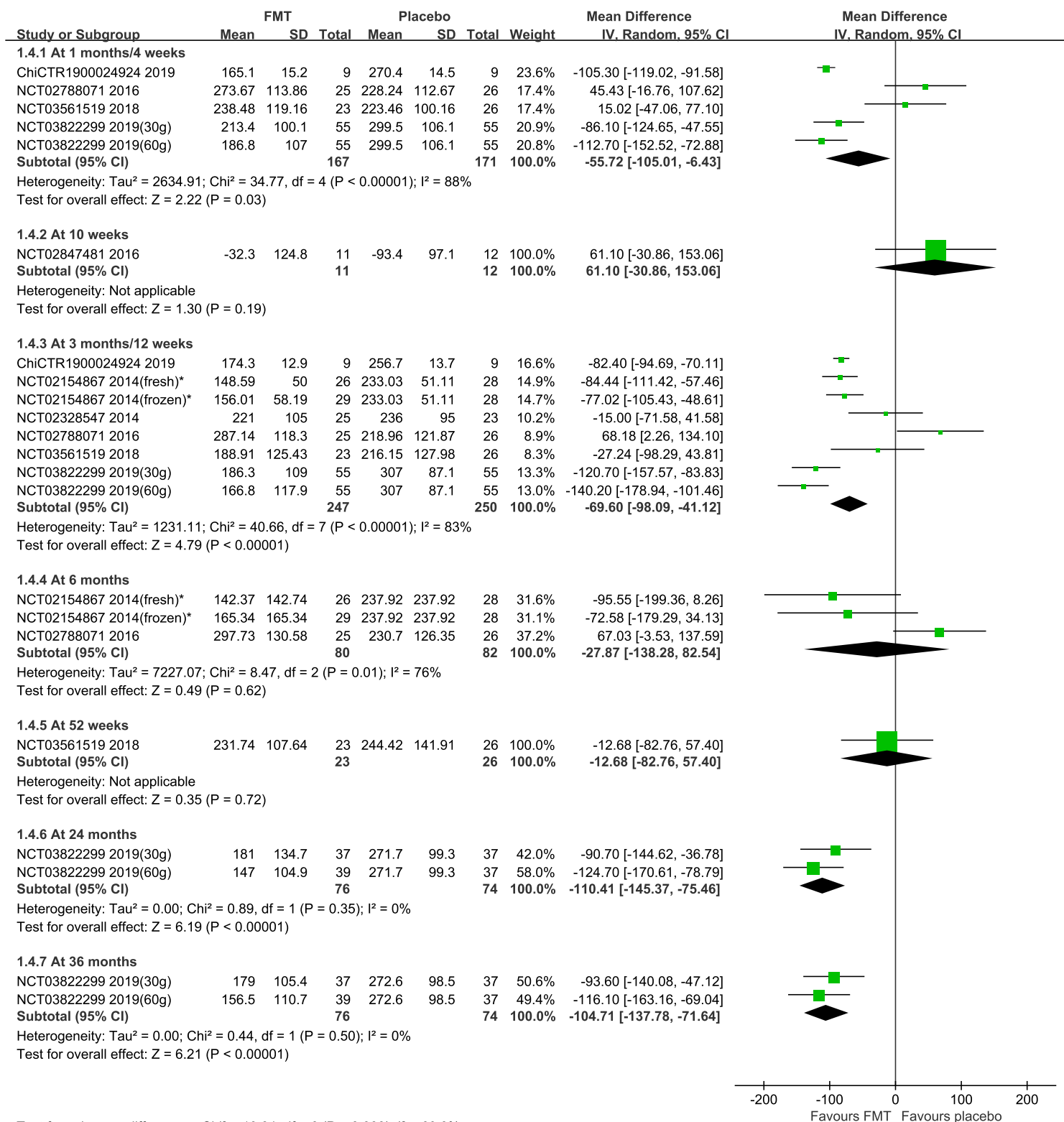

Supplement: Supplementary Figure 1 — Clinical response rate at different times between FMT and placebo groups [file DataSheet_1.zip › Supplementary materials/Supplementary figure 2.pdf]

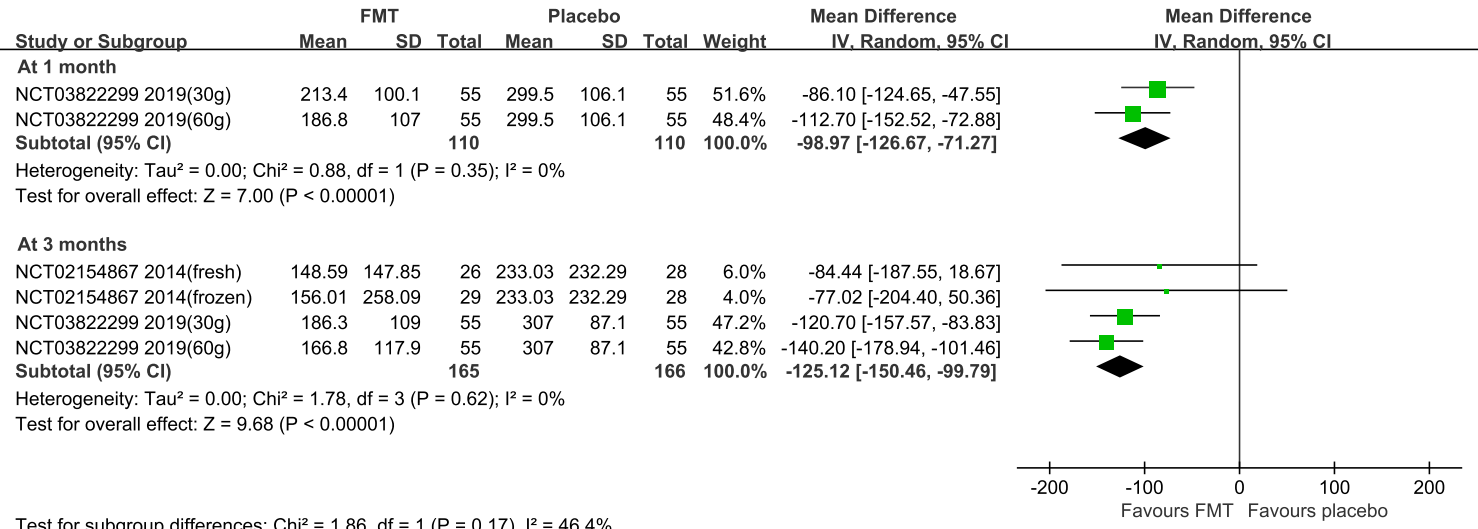

Supplement: Supplementary Figure 1 — Clinical response rate at different times between FMT and placebo groups [file DataSheet_1.zip › Supplementary materials/Supplementary figure 3.pdf]

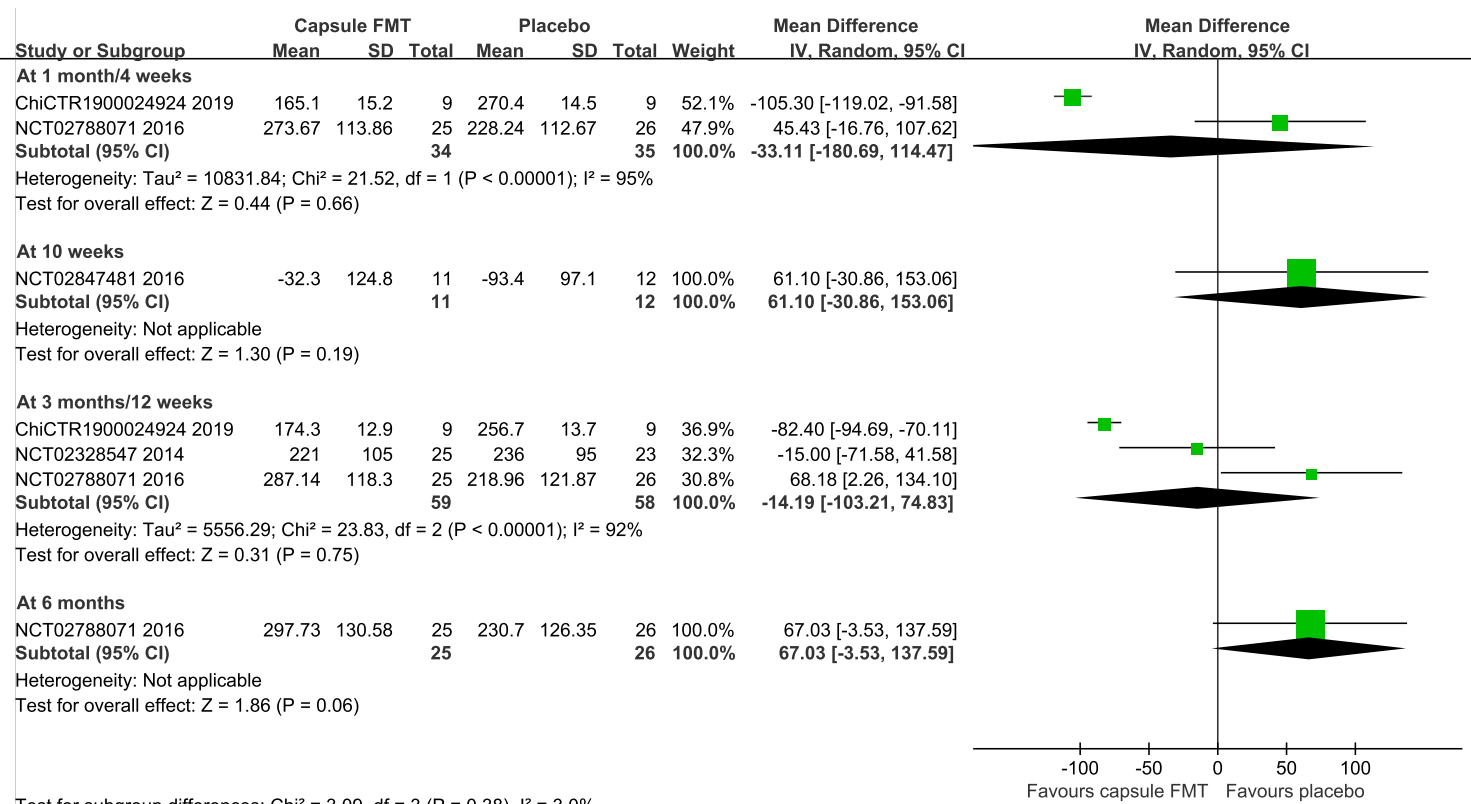

Supplement: Supplementary Figure 1 — Clinical response rate at different times between FMT and placebo groups [file DataSheet_1.zip › Supplementary materials/Supplementary figure 4.pdf]

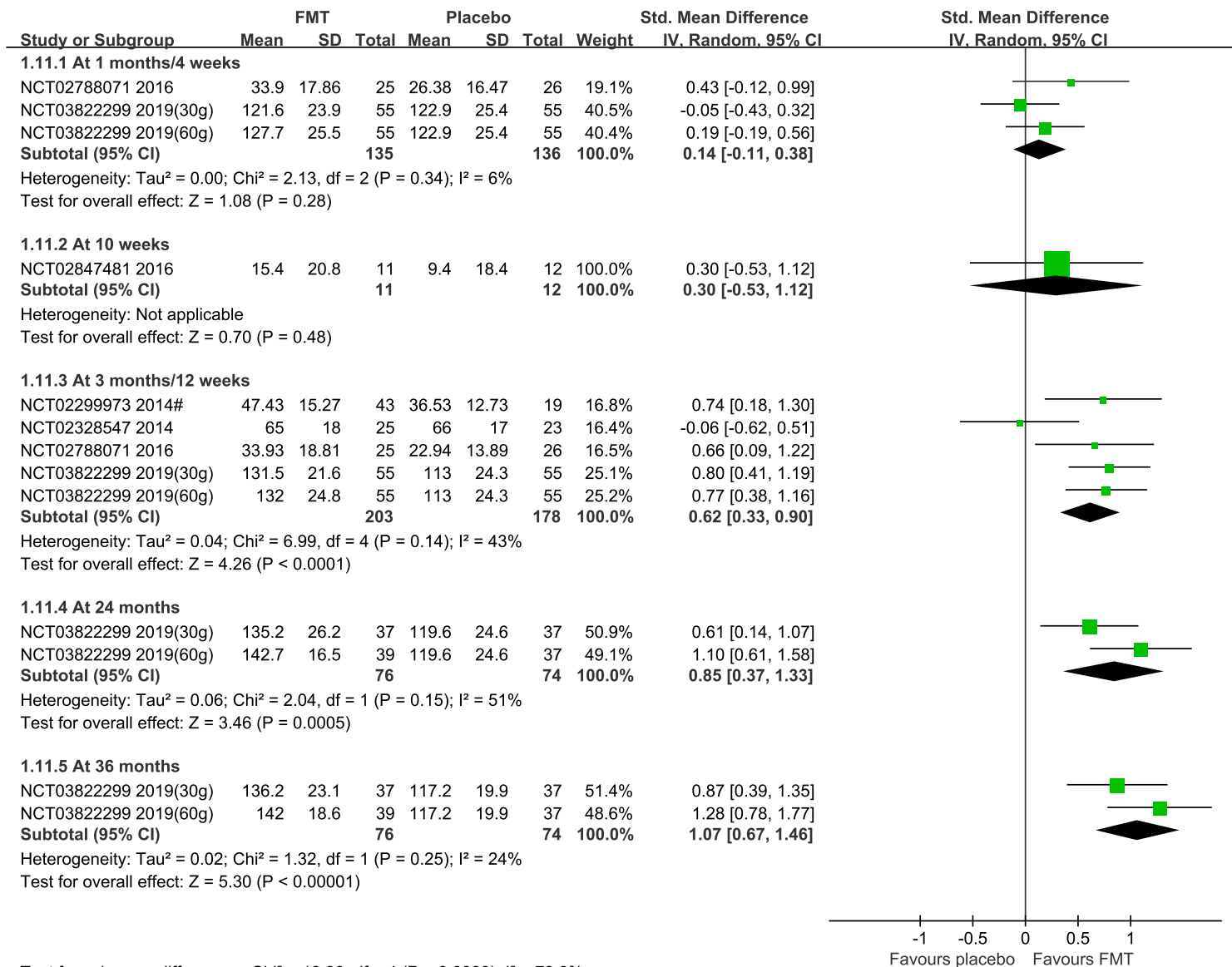

Supplement: Supplementary Figure 1 — Clinical response rate at different times between FMT and placebo groups [file DataSheet_1.zip › Supplementary materials/Supplementary figure 5.pdf]
